# Resolving Interpretation Challenges in Machine Learning Feature Selection With an Iterative Approach in Biomedical Pain Data

**DOI:** 10.1002/ejp.70221

**Published:** 2026-01-26

**Authors:** Jörn Lötsch, André Himmelspach, Dario Kringel

**Affiliations:** ^1^ Faculty of Medicine Goethe University, Institute of Clinical Pharmacology Frankfurt am Main Germany; ^2^ Faculty of Medicine University of Helsinki Helsinki Finland; ^3^ Fraunhofer Institute for Translational Medicine and Pharmacology (ITMP) Frankfurt am Main Germany

**Keywords:** data science, effect sizes, feature selection, knowledge discovery, machine learning, pain research, statistics

## Abstract

**Background:**

Machine learning (ML) is increasingly used to analyse pain‐related data, emphasising how well variables classify individuals, that is, training an algorithm to assign people to predefined groups such as high versus low pain sensitivity, rather than focusing on *p*‐values. A challenge arises when accurate classification persists after removing variables identified as important by feature‐selection methods. This creates uncertainty about which factors are genuinely relevant to the trait of interest, as classification information may still reside in the remaining features.

**Methods:**

An iterative ML framework is presented that repeatedly tests groups of variables, combining two established feature‐selection techniques with several classification algorithms. The approach was applied to three datasets, two assessing pain traits and one artificial, and compared with classical statistical methods, including logistic regression.

**Results:**

The iterative process clarified which variables were truly relevant for classification by assessing whether unselected features could still discriminate individuals. When they could not, selected variables became more interpretable in a biological context. Combining multiple ML approaches improved feature selection, addressed multicollinearity and enhanced robustness across models. Logistic regression sometimes required preselected inputs or missed known relevant variables. Variation in model performance increased interpretive complexity.

**Conclusions:**

ML‐based feature selection broadens methodological options for identifying trait‐relevant variables. Iterating through variable sets supports transparent, replicable inference. ML can help identify variables related to pain traits, but selected features should not be assumed uniquely important. Testing unselected variables remains essential, as their failure to predict outcomes may reflect algorithmic limitations rather than definitive trait exclusivity.

**Significance Statement:**

This study presents an iterative machine learning framework that improves the identification of trait‐relevant features in biomedical pain data. This framework reduces ambiguity in feature selection and clarifies interpretation, helping to distinguish robust, meaningful predictors from coincidental ones. This approach enhances the interpretation and transparency of machine learning analyses in pain research and related biomedical fields.

## 1 Introduction

Machine learning methods are becoming increasingly integrated into biomedical data analysis including pain research (for overview, see e.g., Lötsch et al. 2017; Lötsch et al. 2022), for example when biomarkers are addressed (Sisignano et al. 2019). In machine learning, the focus is less on inference about individual effects of variables and their statistical significance, but more on predictive usefulness, that is, on the utility of variables to enable classification of new data into, for example, healthy versus diseased. This means that variables considered ‘important’ are those that help a trained model make accurate predictions (Lo et al. 2015; Varga et al. 2020). Therefore, how well a feature enables classification, or its classification performance, is used instead of traditional *p*‐values.

Feature selection methods (Guyon 2003) identify the most informative variables from a large set of candidates based on their information content and utility in predictive tasks. These approaches are used not only to define subsets of predictive variables but also to reveal which individual features are most relevant for understanding the underlying biology. This usage assumes that features enabling reliable classification are both informative and biologically meaningful. Consequently, biomedical interpretation typically centres on the selected variables, while non‐selected features are often implicitly regarded as irrelevant and omitted from further analyses.

However, it is not a rare observation that the variables, which an algorithm considers unimportant, can still be used to classify cases correctly. This makes it difficult to decide which variables are truly relevant biologically (Bontonou et al. 2025). We have noted this phenomenon earlier and proposed that the classification power of variables excluded during feature selection (Guyon 2003) should still be considered in biomedical interpretation (Lötsch and Ultsch 2022).

In the present report, we introduce a systematic feature selection and testing framework where classification performance with the unselected features is addressed alongside the selected features, with the aim to identify all features with which classification is possible and finally exclude only those features with which this consistently fails. We will demonstrate that this machine learning‐based framework can identify the key variables driving a trait of interest as well as classical statistics can, and sometimes even substantially better.

Starting with a tailored motivational example, we will apply the framework for determining the clear inclusion or exclusion of variables relevant to the trait of interest, and we will compare these outcomes with classical statistical methods and their potential to provide established and reliable research answers. While this framework is based on machine learning, its focus is placed on interpreting which variables enable classification at all, rather than on maximising classification performance.

## 2 Motivating Example From Pain Research

### 2.1 Pain Thresholds Related Dataset (‘pain_thresholds_sex’)

#### 2.1.1 Original Preprocessed Dataset

A quantitative sensory testing dataset containing pain thresholds from 125 healthy volunteers (69 men and 56 women) was previously published (Doehring et al. 2011). The dataset is freely accessible at https://data.mendeley.com/datasets/9v8ndhctvz/1, and plots of original raw data are shown and freely accessible in figure 1 of Doehring et al. (2011). It includes 11 variables measuring pain thresholds to mechanical stimuli, distinguished as blunt and punctate pressure, as well as thermal stimuli (heat and cold), and electrical stimuli (5 Hz sine wave impulses). Pain thresholds for punctate pressure and heat stimuli were measured again following topical sensitisation with capsaicin cream, while cold thresholds were reassessed after menthol cream application. The dataset also includes the calculated sensitisation effects of capsaicin on pain thresholds to heat or punctate pressure stimuli and the effects of menthol on pain thresholds to cold stimuli. Previous studies found the largest sex differences for pressure pain (Doehring et al. 2011), and classification algorithms trained on blunt pressure pain, alone or combined with punctate pressure or electrical pain, predicted sex with about 80% accuracy (Lötsch et al. 2023). For the present assessments, the preprocessed dataset from the latter publication was used.

#### 2.1.2 Modification to the ‘pain_thresholds_sex’ Dataset

When classification can be achieved using both selected and excluded features, it becomes difficult to interpret the selected features as biologically relevant ones. To explore this issue, we conducted a controlled experiment by duplicating the variable with the largest sex effect, which we later want to predict, the blunt pressure pain threshold, and examined how this modification influenced the results. The rationale was that if only one of the duplicated variables were selected, the other would necessarily end up in the unselected set. This ensured that the unselected subset contained at least one classification‐relevant feature, thereby creating the experimental condition described in the introduction. To account for measurement variability, Gaussian noise was introduced to the pressure threshold values by adding normally distributed random variation (mean = 0) with a standard deviation equal to 20% of the absolute value of each measurement (correlation between original ‘Pressure’ and copied ‘Pressure2’: *r* = 0.98). The analysed dataset was a 125 × 12 matrix (125 cases, 12 variables).

For the machine learning analyses, we reused the same stratified 80/20 split as in the previous work, keeping the original class proportions. The validation set consisted of 20% of the data (14 men and 11 women), and the remaining 80% (55 men and 45 women) was used for feature selection and classifier training (Lötsch et al. 2023). In line with standard practice, prediction was therefore evaluated on data not involved in any model building, analogous to independent replication samples in genetic association studies as an example from which this concept is familiar to pain researchers. Concretely, the training set was used to select features and develop and tune models, while the held‐out validation set was used only for final performance assessment. If a trained model cannot correctly predict class labels (e.g., men versus women, healthy versus diseased), the model is not considered successful, and its components are not regarded as trait‐relevant. Within this framework, a variable is considered relevant if it consistently contributes to successful classification in new data, rather than because it meets a predefined statistical significance threshold. This perspective does not reject statistical inference but instead emphasises predictive usefulness as a complementary criterion for relevance.

For comparative statistical evaluations, unless otherwise indicated, the entire dataset was used as it is standard for classical analyses, that is, training/test and validation subsets were reunited and analysed together.

### 2.2 Machine Learning‐Based Analysis of the Pain Quantitative Sensory Testing Dataset

To analyse the given dataset, we first applied common feature selection methods to identify features that might be biologically relevant. Next, we trained classifiers using only these selected features to evaluate whether they indeed enabled accurate classification. Finally, we also trained classifiers on the unselected features to see if they could achieve similar performance. Feature selection was therefore embedded in an iterative procedure. At each step, selected variables were removed and classification performance was reassessed using the remaining variables. Only features whose removal led to a clear loss of classification performance were retained as finally selected, ensuring that relevance was defined by functional necessity rather than algorithmic preference (Bontonou et al. 2025).

#### 2.2.1 Feature Selection

We applied Least Absolute Shrinkage and Selection Operator (LASSO) regression (Fonti and Belitser 2017) using the ‘glmnet’ R package (https://cran.r‐project.org/package=glmnet; Friedman et al. 2010), which is a regression‐based method that reduces the influence of less informative variables by shrinking their estimated effects toward zero. Variables that retain non‐zero effects after this process are interpreted as contributing more strongly to prediction, while weaker variables are effectively excluded (Friedman et al. 2010; Tay et al. 2023). A binomial logistic model was fitted, and the regularisation parameter was optimised by 5‐fold cross‐validation using the deviance loss function. Features with non‐zero coefficients at the $\lambda_{\min}$ value (excluding the intercept) were retained as selected variables. This procedure ensures that only the most predictive features are kept in the final model and, like Boruta, provides a clear yes/no decision for each feature.

Complementarily, we used the Boruta method (Kursa and Rudnicki 2010), which uses importance scores based on random forests (Breiman 2001), computed from bootstrap samples and out‐of‐bag observations (i.e., data left out of each tree's bootstrap sample), which serve as the basis for comparing original variables with their randomised shadow counterparts, which are randomised versions of these variables. In the present implementation, 100 importance estimation iterations (the default of the R library ‘Boruta’; https://cran.r‐project.org/package=Boruta; Kursa and Rudnicki 2010) were performed using different random seeds. The Boruta algorithm evaluates variable importance by comparing each original feature to randomised versions of itself. Features that consistently outperform these randomised counterparts across repeated analyses are considered informative. This approach aims to identify variables that carry meaningful predictive information beyond random variation. Variables are classified as ‘confirmed’, ‘tentative’ or ‘rejected’, with only ‘confirmed’ variables accepted as selected features. This approach provides a robust statistical evaluation of feature importance, reducing the risk of overfitting and enhancing the reliability of selected features. As with the LASSO implementation, it ultimately provides a clear yes/no decision about each feature.

**FIGURE 1:**
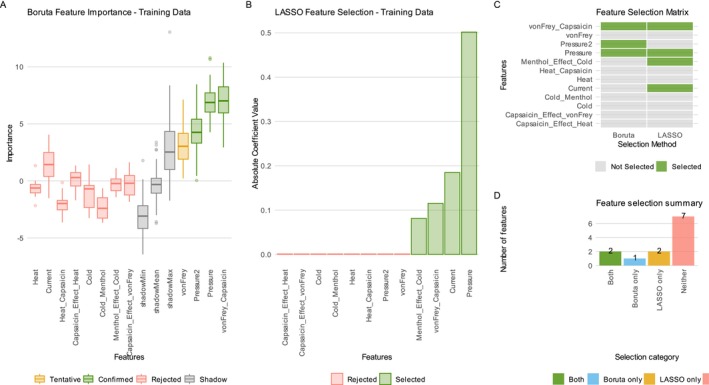
Pain thresholds dataset: Feature selection results using Boruta and LASSO methods. The first two panels show Boruta importance scores as boxplots and LASSO absolute coefficient values as barplots, with features coloured by selection status (‘Confirmed’ or ‘Selected’ in green, ‘Tentative’ in orange and ‘Rejected’ in salmon). The third panel presents a heat map matrix illustrating feature selection overlap between Boruta and LASSO, with green tiles indicating selection by each method. The fourth panel summarises the counts of features uniquely selected by each method, selected by both methods or rejected by both.

Both methods highlighted partly, but not completely, the same features. The selection emphasised mechanical pain as a most distinctive quantitative sensory measure related to sex (Figure 1), which agrees with prior findings in the same dataset (Lötsch et al. 2023). Boruta selected the two blunt‐pressure pain‐related variables (original and noise‐perturbed copy) and pain thresholds to punctate pressure after capsaicin sensitisation. LASSO identified only one of the two blunt‐pressure pain threshold variables, the pain thresholds to punctate pressure after sensitisation (von Frey hairs), the pain threshold to 5 Hz electrical sine wave stimulus without prior sensitisation, and, as an additional finding, the menthol effect against cold stimuli.

#### 2.2.2 Classification Tasks and Model Evaluation

Using the selected features above, we trained machine learning classifiers including random forests, support vector machines (SVM) (Cortes and Vapnik 1995), k‐nearest neighbours (Cover and Hart 1967) and C5.0 decision trees (Quinlan 1986), but also logistic regression, with the 80% training/test subset (see above), to assign a study case to its correct sex. Classification performance was evaluated on the 20% validation subset, using balanced accuracy as a robust measure insensitive to class size imbalance (Brodersen et al. 2010). This was done in a 100 runs set‐up as a Monte Carlo (Metropolis and Ulam 1949) cross‐validation framework, which repeatedly evaluates models on many random splits to obtain stable performance estimates. Successful classification was defined as median balanced accuracy > 0.5 with confidence intervals (CI) excluding chance, that is, the lower border of the non‐parametric 95% CI also had to be greater than the 0.5 chance level.

The classification analyses were implemented in R using several libraries. The ‘randomForest’ package was employed for training and tuning random forest (RF) classifiers (https://cran.r‐project.org/package=randomForest; Liaw and Wiener 2002). The base R ‘stats’ package was used for binomial logistic regression via the ‘glm’ function, while the ‘nnet’ package handled multinomial logistic regression models (https://cran.r‐project.org/package=nnet; Venables and Ripley 2002). The ‘caret’ package served as a central framework for model training, cross‐validation and hyperparameter tuning, including k‐nearest neighbours (KNN) and Support Vector Machines (SVM) through its unified ‘train()’ interface (https://cran.r‐project.org/package=caret; Kuhn 2018). SVMs were trained using the ‘svmRadial’ method provided via the ‘kernlab’ backend (https://cran.r‐project.org/package=kernlab; Karatzoglou et al. 2004), and KNN classifiers used caret's built‐in ‘knn’ method. Decision tree models based on the C5.0 algorithm were fitted using the ‘C50’ package (https://cran.r‐project.org/package=C50; Kuhn and Quinlan 2018). Model evaluation metrics, including the area under the ROC curve (AUC), were obtained with the ‘pROC’ package (https://cran.r‐project.org/package=pROC; Robin et al. 2011), and confusion matrices and balanced accuracy were computed using functions from ‘caret’.

As expected, classification success varied among classifiers and feature sets (all variables or selected features by Boruta or LASSO; Table 1). Notably, the C5.0 tree classifier performed poorly, and the 95% confidence interval of the balanced classification accuracy always included the guessing level of 0.5, indicating failure. However, one successful classification algorithm is sufficient for the sake of this motivating example. Importantly, classification was also possible in some scenarios with unselected features, reproducing the setting described in the introduction and reported in (Bontonou et al. 2025). This observation, particularly with LASSO‐selected features that missed redundant or artificially added variables, served as the starting point for developing the feature selection and evaluation framework detailed in the next chapter.

**TABLE 1:** Pain thresholds dataset: Classification performance based on 100 repeated runs and feature selection pipeline results.

Dataset	Features	RF_BA	LR_BA	KNN_BA	C50_BA	SVM_BA	Classification_Success	Phase	Features_Used
All_Features	12	0.641 [0.485, 0.788]	0.750 [0.634, 0.873]	0.600 [0.479, 0.731]	0.510 [0.374, 0.770]	0.635 [0.467, 0.823]	1	Phase_0_Full	Heat; Pressure; Current; Heat_Capsaicin; Capsaicin_Effect_Heat; Cold; Cold_Menthol; Menthol_Effect_Cold; vonFrey; vonFrey_Capsaicin; Capsaicin_Effect_vonFrey; Pressure2
Boruta_Selected	3	0.641 [0.468, 0.794]	0.742 [0.555, 0.895]	0.646 [0.446, 0.821]	0.503 [0.359, 0.779]	0.644 [0.467, 0.799]	1	Phase_0_Full	Pressure; vonFrey_Capsaicin; Pressure2
Boruta_Rejected	9	0.520 [0.389, 0.709]	0.586 [0.434, 0.742]	0.495 [0.258, 0.669]	0.535 [0.366, 0.766]	0.568 [0.450, 0.677]	0	Phase_0_Full	Heat; Current; Heat_Capsaicin; Capsaicin_Effect_Heat; Cold; Cold_Menthol; Menthol_Effect_Cold; vonFrey; Capsaicin_Effect_vonFrey
LASSO_Selected	4	0.667 [0.492, 0.756]	0.742 [0.631, 0.849]	0.662 [0.497, 0.790]	0.521 [0.379, 0.776]	0.708 [0.558, 0.817]	1	Phase_0_Full	Pressure; Current; Menthol_Effect_Cold; vonFrey_Capsaicin
LASSO_Rejected	8	0.542 [0.352, 0.700]	0.708 [0.550, 0.854]	0.540 [0.389, 0.667]	0.538 [0.350, 0.843]	0.634 [0.487, 0.780]	1	Phase_0_Full	Heat; Heat_Capsaicin; Capsaicin_Effect_Heat; Cold; Cold_Menthol; vonFrey; Capsaicin_Effect_vonFrey; Pressure2
Boruta_LASSO_Selected	5	0.667 [0.530, 0.788]	0.742 [0.631, 0.849]	0.687 [0.562, 0.792]	0.521 [0.374, 0.792]	0.729 [0.508, 0.847]	1	Phase_0_Full	Pressure; vonFrey_Capsaicin; Pressure2; Current; Menthol_Effect_Cold
Boruta_LASSO_Rejected	7	0.375 [0.232, 0.494]	0.394 [0.275, 0.513]	0.484 [0.341, 0.616]	0.423 [0.356, 0.505]	0.457 [0.320, 0.608]	0	Phase_0_Full	Heat; Heat_Capsaicin; Capsaicin_Effect_Heat; Cold; Cold_Menthol; vonFrey; Capsaicin_Effect_vonFrey
Only_high_correlated	1	0.679 [0.541, 0.813]	0.772 [0.677, 0.897]	0.750 [0.625, 0.854]	0.669 [0.480, 0.882]	0.742 [0.240, 0.859]	1	Phase_0_Full	Pressure2
All_Features	3	0.674 [0.564, 0.800]	0.778 [0.638, 0.870]	0.700 [0.589, 0.810]	0.625 [0.500, 0.804]	0.753 [0.547, 0.872]	1	Phase_3_Final_Selected	Pressure2; Pressure; Current
Boruta_Selected	1	0.652 [0.497, 0.773]	0.772 [0.677, 0.897]	0.737 [0.551, 0.854]	0.571 [0.489, 0.800]	0.742 [0.250, 0.849]	1	Phase_3_Final_Selected	Pressure
Boruta_Rejected	2	0.637 [0.529, 0.749]	0.771 [0.631, 0.889]	0.692 [0.540, 0.823]	0.650 [0.489, 0.859]	0.753 [0.271, 0.850]	1	Phase_3_Final_Selected	Pressure2; Current
LASSO_Selected	2	0.634 [0.462, 0.738]	0.778 [0.638, 0.870]	0.729 [0.634, 0.800]	0.604 [0.489, 0.804]	0.751 [0.562, 0.854]	1	Phase_3_Final_Selected	Pressure; Current
LASSO_Rejected	1	0.679 [0.541, 0.813]	0.772 [0.677, 0.897]	0.750 [0.625, 0.854]	0.669 [0.480, 0.882]	0.742 [0.240, 0.859]	1	Phase_3_Final_Selected	Pressure2
Boruta_LASSO_Selected	2	0.634 [0.462, 0.738]	0.778 [0.638, 0.870]	0.729 [0.634, 0.800]	0.604 [0.489, 0.804]	0.751 [0.562, 0.854]	1	Phase_3_Final_Selected	Pressure; Current
Boruta_LASSO_Rejected	1	0.679 [0.541, 0.813]	0.772 [0.677, 0.897]	0.750 [0.625, 0.854]	0.669 [0.480, 0.882]	0.742 [0.240, 0.859]	1	Phase_3_Final_Selected	Pressure2
Only_high_correlated	1	0.652 [0.497, 0.773]	0.772 [0.677, 0.897]	0.737 [0.551, 0.854]	0.571 [0.489, 0.800]	0.742 [0.250, 0.849]	1	Phase_3_Final_Selected	Pressure
All_Features	9	0.398 [0.250, 0.550]	0.394 [0.275, 0.513]	0.454 [0.308, 0.616]	0.455 [0.349, 0.541]	0.459 [0.306, 0.558]	0	Phase_3_Final_Rejected	Heat; Heat_Capsaicin; Capsaicin_Effect_Heat; Cold; Cold_Menthol; Menthol_Effect_Cold; vonFrey; vonFrey_Capsaicin; Capsaicin_Effect_vonFrey
Boruta_Selected	2	0.487 [0.333, 0.639]	0.482 [0.354, 0.598]	0.465 [0.333, 0.595]	0.455 [0.349, 0.541]	0.424 [0.339, 0.545]	0	Phase_3_Final_Rejected	vonFrey; vonFrey_Capsaicin
Boruta_Rejected	7	0.424 [0.291, 0.559]	0.495 [0.360, 0.625]	0.500 [0.360, 0.699]	0.500 [0.450, 0.526]	0.500 [0.381, 0.615]	0	Phase_3_Final_Rejected	Heat; Heat_Capsaicin; Capsaicin_Effect_Heat; Cold; Cold_Menthol; Menthol_Effect_Cold; Capsaicin_Effect_vonFrey
LASSO_Selected	3	0.458 [0.313, 0.634]	0.447 [0.348, 0.568]	0.500 [0.336, 0.651]	0.455 [0.347, 0.541]	0.439 [0.333, 0.566]	0	Phase_3_Final_Rejected	Menthol_Effect_Cold; vonFrey; vonFrey_Capsaicin
LASSO_Rejected	6	0.439 [0.293, 0.562]	0.495 [0.360, 0.625]	0.402 [0.292, 0.551]	0.500 [0.459, 0.500]	0.500 [0.344, 0.623]	0	Phase_3_Final_Rejected	Heat; Heat_Capsaicin; Capsaicin_Effect_Heat; Cold; Cold_Menthol; Capsaicin_Effect_vonFrey
Boruta_LASSO_Selected	3	0.479 [0.303, 0.615]	0.447 [0.348, 0.568]	0.500 [0.336, 0.651]	0.455 [0.347, 0.541]	0.439 [0.333, 0.566]	0	Phase_3_Final_Rejected	vonFrey; vonFrey_Capsaicin; Menthol_Effect_Cold
Boruta_LASSO_Rejected	6	0.439 [0.293, 0.562]	0.495 [0.360, 0.625]	0.402 [0.292, 0.551]	0.500 [0.459, 0.500]	0.500 [0.344, 0.623]	0	Phase_3_Final_Rejected	Heat; Heat_Capsaicin; Capsaicin_Effect_Heat; Cold; Cold_Menthol; Capsaicin_Effect_vonFrey

*Note:* Median balanced accuracy (BA) with non‐parametric 95% confidence intervals from 100 bootstrap iterations using five classifiers: Random Forest (RF), Logistic Regression (LR), K‐nearest neighbours (KNN), C5.0 decision trees (C50) and support vector machines (SVM). Classification_Success indicates whether any classifier achieved a lower confidence interval boundary exceeding 0.5 (1 = success, 0 = failure). Phase_0_Full: Initial screening of all feature combinations from the complete 12‐feature dataset, including the full set and subsets identified by Boruta and LASSO feature selection methods, along with their unions, intersections and rejected features. Phase_3_Final_Selected: The minimal feature set (Pressure2, Pressure, Current) after backward elimination and rescue procedures, tested to verify it cannot be further reduced while maintaining classification success. Phase_3_Final_Rejected: The 9 excluded features tested individually and in all combinations resulting from the various feature selection methods; none achieved classification success, confirming their exclusion. Features_Used lists the specific features in each subset (semicolon‐separated). The final outcome represents a minimal irreducible set of 3 features that maintain classification performance, with the remaining 9 features confirmed as unable to achieve classification in any tested combination. ‘Only_high_correlated’ refers to the pressure‐pain‐related variables that were assigned to the unselected subset by the respective feature selection method (here, LASSO).

### 2.3 Summary of the Introductory Example

This example illustrates challenges that may arise in pain research, and biomedical studies more generally, when topically interpreting traits based on machine learning selected variables. Quantitative sensory testing measures, especially those linked to mechanical and electrical pain thresholds, were identified as useful for sex classification. However, training with unselected features enabled some algorithms to perform sex assignment above chance. Although feature importance scores provided an interpretable ranking, the overall interpretation was less definitive than standard statistical analysis, where a specific *p*‐value seems to offer a clear criterion for relevance. This motivated further experiments to resolve the uncertainty around feature interpretability in machine learning‐based analyses.

## 3 Methods

A general feature selection and testing framework was developed to resolve the ambiguity that arises when machine learning selects features enabling successful classification, yet unselected features still contain relevant information for the classification task. While the primary focus was on enhancing interpretation within the machine learning context, the approach also included a comparative evaluation of standard statistical methods with respect to achieving a clear and comprehensive identification of trait‐relevant features.

### 3.1 Computing Environment

All analyses were implemented in the R language (Ihaka and Gentleman 1996) using R version 4.5.1 for Linux (https://CRAN.R‐project.org/). Coding was performed within the PyCharm integrated development environment (version 2025.2.0.1 Professional Edition, JetBrains, Prague, Czech Republic) with the AI Assistant plugin (version 252.23892.530, https://plugins.jetbrains.com/plugin/22282‐jetbrains‐ai‐assistant). Relevant code including instructions for use is available at the project's web site at https://github.com/JornLotsch/pain‐threshold‐analysis. The R packages used are mentioned alongside the method description. Plotting was done using the R packages ‘ggplot2’ (Wickham 2009) and ‘plotly’ (Sievert 2020). To verify selected results and guard against coding errors, some analyses were occasionally performed using SPSS (Version 27 for Linux, IBM Corp., Armonk, NY, USA), a widely used statistical software package in the social sciences. The computations were run on an AMD Ryzen Threadripper PRO 7985WX 64‐Cores (Advanced Micro Devices Inc., Santa Clara, CA, USA) computer running Ubuntu Linux 24.04.3 LTS (Canonical, London, UK).

### 3.2 Machine Learning‐Based Analysis

#### 3.2.1 Dataset Preparation

For SVM and kNN classifiers, data were preprocessed by centering and scaling. For the other models, including tree‐based random forests or C5.0, as well as logistic regression, this is not necessary.

To ensure that performance estimates reflected true generalisation rather than reuse of information, data were split into two stages. One subset was used exclusively for model development, while a separate subset was reserved for final evaluation. First, a fixed validation sample of 20% of the total dataset was partitioned before any feature selection or model training. This ensured that the performance evaluation was conducted on completely unseen data and prevented information leakage from the validation set into the training or feature selection processes.

Second, to quantify variability and construct confidence intervals for the balanced accuracy metric, repeated random subsampling was performed within the training and validation samples. Specifically, for each of 100 iterations (see below), models were trained on a randomly selected 80% subset of the training data and evaluated on a randomly selected 80% subset of the fixed validation data. Different random seeds generated independent splits. This Monte Carlo cross‐validation framework assesses variability in model performance induced by different training and validation subsets. Empirical non‐parametric confidence intervals derived from this procedure provide a robust, nonparametric quantification of uncertainty in model performance, accommodating potential complexities and peculiarities in the data distribution beyond standard parametric assumptions (Kohavi 1995). The chosen partition proportions balance the need to maintain sufficiently large training subsets for model stability against the requirement for adequate validation sample sizes to reliably estimate performance uncertainty. This approach is particularly suited to datasets of moderate size, typically ranging from about 100 to 150 cases.

#### 3.2.2 Iterative Feature Selection and Classification Framework

**FIGURE 2:**
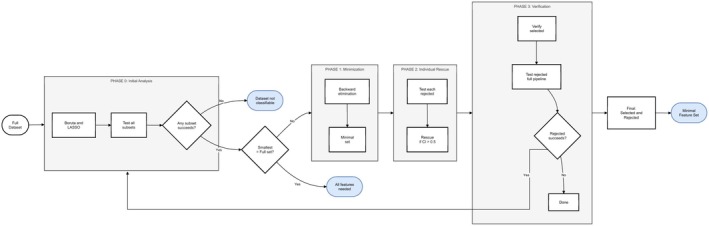
Proposed workflow for feature selection and testing, designed to identify the relevant features while ensuring that the unselected features do not contain information that could enable the successful training of any of the included classification algorithms.

To identify the truly minimal sufficient feature set for classification, the analytical framework was organised into four phases minimising systematically redundancy while ensuring no valuable features are overlooked (Figure 2). First, an initial analysis identified candidate features and baseline performance. Second, selected features were systematically removed and models were retrained to test whether classification could still be achieved. Third, features that failed to support classification were examined individually to confirm their lack of contribution. Together, these phases allowed a structured evaluation of both selected and rejected features.

##### 3.2.2.1 Phase 0: Initial Analysis

In this work, we chose LASSO and Boruta as feature selection methods as described in the introductory example. In the case of Boruta, features were deemed relevant when they appeared in the ‘confirmed’ group. LASSO features were deemed relevant when the respective LASSO feature coefficient was larger than 0. In Phase 0, Boruta and LASSO were applied to the full training dataset (see above), that is, the training data with all variables.

Then, multiple feature subset combinations (all features, Boruta‐selected, LASSO‐selected, Boruta‐rejected, LASSO‐rejected and their unions/intersections) were tested whether they suffice to train a classifier that successfully assigns a new case, unseen during training, to the correct class, for example, men or women in the introductory pain thresholds dataset. The five classifiers already chosen for the introductory example were used again, that is, random forests, logistic regression, k‐nearest neighbours, C5.0 decision tree and support vector machines. For each combination of features, classifiers were tuned by grid search for suitable hyperparameters (Lötsch and Mayer 2022), such as the number of trees in random forests or the number of neighbours in kNN, among others.

If none of these feature subsets achieved classification success, defined as any classifier exhibiting a lower bound of the 95% confidence interval for balanced accuracy exceeding 0.5, based on 100 Monte‐Carlo cross‐validation iterations as described above, the algorithm terminated and the dataset was deemed not classifiable, as even the complete feature set failed to produce reliable predictions. If at least one subset was successful, the smallest successful subset was identified. If this smallest subset was identical to the full feature set, all features were considered necessary and the procedure stopped; otherwise, this smallest successful subset was passed to Phase 1.

##### 3.2.2.2 Phase 1: Minimisation of the Feature Set

From the successful Phase 0 results, the smallest feature set that achieved classification success served as the starting point for minimisation. Phase 1 employed backward elimination on this set, sequentially testing the removal of each feature and permanently removing those whose absence still maintained classification success. This process was repeated until no further features could be removed without loss of classification success, yielding a minimal feature set on which any classifier could still be trained to successfully classify new unseen cases. This is in principle the same as reported recently, although in technically different implementation, that is, reducing machine learning‐based feature sets to their minimum informative size (Lötsch and Ultsch 2023b). However, this bare minimum can leave out features that also allow classification, so the present report does not stop at this stage and uses the resulting minimal set as the basis for Phase 2.

##### 3.2.2.3 Phase 2: Individual Rescue of Rejected Features

In Phase 2, a rescue mechanism was implemented to avoid incorrectly discarding potentially relevant variables. Each rejected feature was tested individually to determine whether it could still support classification on its own. Features that consistently failed in this test were considered unlikely to carry independent predictive information. Each rejected feature was used alone to train and evaluate the classifiers; any feature demonstrating classification success (lower 95% CI for balanced accuracy > 0.5) was rescued and reintegrated into the selected feature set. This phase is essential because feature selection procedures can discard individually strong predictors due to redundancy masking, interaction effects or stochastic variability. For example, a feature ‘A’ may have independent classification ability (CI > 0.5) but be rejected if features ‘B’ and ‘C’ together form a stronger combined predictor. Omitting Phase 2 would risk losing scientifically valuable features (‘A’) that possess genuine independent predictive power but were overshadowed during Phase 0 or Phase 1. This exactly targets the problem raised in the introduction, namely that features not having passed feature selection can, when used alone, still classify (Bontonou et al. 2025).

##### 3.2.2.4 Phase 3: Verification and Analysis of the Rejected Set

After Phases 0–2, the feature set is expected to contain all features that enable successful classification (including rescued ones), while rejected features should not. Phase 3 verifies this by jointly assessing the final selected and rejected sets using the same logic as in Phase 0. Although this step may be partially redundant given the preceding phases, it provides an explicit and conservative safeguard and ensures that nothing has been missed in the precedent phases. First, the selected feature set (including all rescued variables) was re‐evaluated to confirm continued classification success by running all feature selectors on the selected features and then running all classifiers on the complete group and across all subsets of unions and intersections. Next, the rejected feature set (all remaining features) is tested in the same way. If no classifier on any subset achieved performance over pure chance, the procedure terminated with the final partition.

However, if at least one classifier on one subset achieved classification success, the full pipeline was rerun on this subset: Boruta and LASSO were reapplied, and all resulting combinations tested. Features selected by either method underwent individual testing; those showing independent success (lower CI > 0.5) were rescued and added to the selected set. Verification was then repeated iteratively until the rejected set no longer classified successfully. In rare cases where the rejected set retained group‐level success but neither Boruta nor LASSO identified features, and no individual feature classified alone, the framework issued a warning, flagging these features as potentially informative only through complex interactions undetectable by both methods.

#### 3.2.3 Model‐Agnostic Implementation

Importantly, this framework does not depend on any single classification algorithm. Instead, it evaluates whether conclusions remain consistent across different modelling approaches, reducing the risk that results are driven by the idiosyncrasies of a particular method. A step‐by‐step pseudocode description is provided in Box 1, specifying only the required inputs and decision rules (feature subsets, performance metrics and confidence thresholds) but not any particular algorithms. Any combination of feature selection methods (e.g., filter, wrapper, embedded) and classifiers can be plugged into Phases 0–3, provided they yield comparable performance estimates and confidence intervals. In this sense, Boruta, LASSO and the current set of classifiers serve as concrete examples rather than fixed components, and researchers may substitute or extend them according to the specifics of their data and scientific question.

BOX 1 **Pseudocode of the iterative feature selection and rejection algorithm. The procedure takes as input a dataset with features and target Y, a set of feature selection methods, and a set of classifiers, and outputs sets of selected and rejected features under the constraint that rejected features do not support successful classification (i.e., classifier performance remains at chance level).**

**Table:** 

Algorithm					
	**Input:** Dataset $D=\left\{X_{1},\ldots ,X_{J},Y\right\}$ with features $X_{j}$ and target Y K many feature selectors $F=\left\{f_{1},\ldots ,f_{K}\right\}$ C many Classifiers $C=\left\{c_{1},\ldots ,c_{C}\right\}$				
	**Output:** Set of selected and rejected features, while confirming that rejected features do not enable classification.				
1:	selected←∅				
2:	$\text{rejected}\leftarrow \left\{X_{1},\ldots ,X_{J}\right\}$				
3:	Current considered features $F_{\mathrm{cur}}\leftarrow \left\{X_{1},\ldots ,X_{J}\right\}$				
4:	**WHILE** $F_{\mathrm{cur}}$ changes **DO**				
5:		# Phase 0			
6:		Build dataset $D_{\text{current}}$ with features $F_{\mathrm{cur}}$ and target Y			
7:		Apply all feature selectors fϵF to $D_{\text{current}}$			
8:		Generate *subsets* of features with: all features, all selected by a feature selector and all possible unions and intersections between those			
9:		*successful_subsets* ←∅			
10:		**FOR EACH** *subset* ∈ *subsets* **DO**			
11:			Build dataset $D_{\text{subset}}$ with features from subset and target Y		
12:			Tune and evaluate all classifiers on $D_{\text{subset}}$ via Monte‐Carlo Cross Validation and mark subset as successful if any classifier's lower confidence interval exceeds 0.5		
13:			**IF** *subset* marked as successful **THEN** *successful_subsets* ← *successful_subsets* ∪ *subset*		
14:		**END FOR**			
15:		**IF** *successful_subsets* ←∅ **THEN RETURN** *selected, rejected*			
16:		Determine the smallest subset in *successful_subsets* and save in *smallest*			
17:		**IF** *smallest* $=F_{\mathrm{cur}}$ **THEN**			
18:			selected←selected∪smallest		
19:			rejected←rejected∖smallest		
20:			**RETURN** *selected, rejected*		
21:		**END IF**			
22:					
23:		# Phase 1			
24:		*minimal* ←smallest			
25:		changed←True			
26:		**WHILE** *changed* = *True* **DO**			
27:			changed←False		
28:			**FOR EACH** feature x∈ *minimal* **DO**		
29:				current← *minimal* $\setminus \left\{x\right\}$	
30:				Build dataset $D_{\text{current}}$ with features from current and target Y	
31:				Tune and evaluate all classifiers on $D_{\text{current}}$ via Monte‐Carlo Cross Validation and mark *current* as successful if any classifier's lower confidence interval exceeds 0.5	
32:				**IF** *current* marked as successful **THEN**	
33:					*minimal* ←current
34:					changed←True
35:					**BREAK**
36:				**END IF**	
37:			**END FOR**		
38:		**END WHILE**			
39:					
40:		# Phase 2			
41:		$\text{selecte}d_{\mathrm{cur}}\leftarrow$ *minimal*			
42:		$\text{rejecte}d_{\mathrm{cur}}\leftarrow F_{\mathrm{cur}}\setminus$ *minimal*			
43:		**FOR EACH** $x\in \text{rejecte}d_{\mathrm{cur}}$ **DO**			
44:			Build Dataset $D_{x}$ with only feature *x* and target Y		
45:			Tune and evaluate all classifiers on $D_{x}$ via Monte‐Carlo Cross Validation and mark *x* as successful if any classifier's lower confidence interval exceeds 0.5		
46:			**IF** *x* marked as successful **THEN** $\text{selecte}d_{\mathrm{cur}}\leftarrow \text{selecte}d_{\mathrm{cur}}\cup \left\{x\right\}$		
47:		**END FOR**			
48:		$\text{selected}\leftarrow \text{selected}\cup \text{selecte}d_{\mathrm{cur}}$			
49:		rejected←rejected∖selected			
50:					
51:		# Phase 3			
52:		Generate all candidate subsets $S_{\text{selected}}$ from *selected*, including all features and all unions and intersections from all features selected by feature selectors f∈F			
53:		**FOR** each $\text{subset}\in S_{\text{selected}}$ **DO**			
54:			Build Dataset $D_{\text{subset}}$ with features from subset and target Y		
55:			Tune and evaluate all classifiers on $D_{\text{subset}}$ via Monte‐Carlo Cross Validation and mark subset as successful if any classifier's lower confidence interval exceeds 0.5		
56:		**END FOR**			
57:		**IF** no $\text{subset}\in S_{\text{selected}}$ is marked **THEN RETURN** an error			
58:		Generate all candidate subsets $S_{\text{rejected}}$ from *rejected*, including all features and all unions and intersections from all features selected by feature selectors f∈F			
59:		**FOR** each $\text{subset}\in S_{\text{rejected}}$ **DO**			
60:			Build Dataset $D_{\text{subset}}$ with features from subset and target Y		
61:			Tune and evaluate all classifiers on $D_{\text{subset}}$ via Monte‐Carlo Cross Validation and mark subset as successful if any classifier's lower confidence interval exceeds 0.5		
62:		**END FOR**			
63:		**IF** no $\text{subset}\in S_{\text{rejected}}$ is marked as successful **THEN RETURN** *selected, rejected*			
64:		**ELSE IF** no x∈rejected was selected by any feature selector during Phase 0 **AND** no x∈rejected was marked successful individually during Phase 2 **THEN**			
65:			**RETURN** *selected, rejected* and a warning that flags *rejected* features as potentially informative		
66:		**END IF**			
67:		$F_{\mathrm{cur}}\leftarrow \text{rejected}$			
68:	**END WHILE**

### 3.3 Comparative Statistical Analyses

#### 3.3.1 Univariate Statistical Tests

Univariate methods assessed each variable independently for association with the binary outcome. Effect size measures, such as Cohen's *d* (Cohen 1992), were used to quantify feature importance and guide selection for further analysis. Statistical significance of group differences was evaluated using *t*‐tests (Student 1908).

#### 3.3.2 Multivariate Regression Modelling

##### 3.3.2.1 Iterative Modelling

Binary logistic regression evaluated the joint effect of predictors on the binary outcome. Candidate variables suspected of multicollinearity or redundancy, such as derived or proxy variables, were identified based on prior exploratory insights. Logistic regression models were systematically constructed across all possible subsets of these candidate variables, enabling evaluation of variable stability, predictive power and the impact of feature dependencies.

##### 3.3.2.2 Diagnostics

Model diagnostics were conducted to detect potential estimation issues such as aliasing and linear dependencies among predictors. Multicollinearity was quantified using variance inflation factors (VIF; Marquardt 1970), which were calculated using the R package ‘car’ (Fox and Weisberg 2011). VIF values above 10 signalled problematic collinearity. Predictors causing estimation failures or inconsistent significance patterns were annotated.

##### 3.3.2.3 Penalised Regression

Penalised logistic regression methods were applied to address overfitting and multicollinearity when standard logistic regression yielded uniformly non‐significant *p*‐values across all predictors (Hoerl and Kennard 1970; Tibshirani 1996; Zou and Hastie 2005). Ridge (L2 penalty), lasso (L1 penalty) and elastic net (combined L1 + L2 penalty, with *α* = 0.5) regressions were implemented using the ‘glmnet’ R package, with the regularisation parameter λ tuned separately for each penalty type. Five‐fold cross‐validation was used to select the optimal λ value, defined as the $\lambda_{\min}$ that minimised cross‐validated deviance for the corresponding model. Variable selection was defined as predictors with non‐zero coefficients at $\lambda_{\min}$ for lasso and elastic net models, and predictors with absolute coefficient magnitude exceeding 0.05 at $\lambda_{\min}$ for ridge regression.

### 3.4 Additional Datasets

In addition to the introductory pain threshold dataset, which was evaluated further to address the remaining issues from the motivation example introduction, further datasets were investigated.

#### 3.4.1 Psoriatic Arthritis Dataset (‘PsA DAS28‐CRP’)

To evaluate the iterative feature selection and classification framework, a rheumatoid arthritis‐related dataset was included from a previously published analysis (Rischke et al. 2023) and plots of original raw data are shown and freely accessible in Figure 2 of that cited paper. The dataset contains clinical records from 80 adults diagnosed with psoriatic arthritis (44 women, 36 men; aged 25–79 years) who presented to or were referred for evaluation at a rheumatology department. For each patient, swelling and tenderness were assessed separately in 28 joints, including bilateral shoulders, elbows, wrists, knees and all metacarpophalangeal and proximal interphalangeal joints of the fingers. These 56 joint‐specific variables were supplemented by blood C‐reactive protein concentration and patient self‐rated global health assessments. Disease activity was then graded using the DAS28‐CRP scoring system, which integrates tender and swollen joint counts, CRP levels and general health assessment into a composite disease activity index (Mease 2011; Singh et al. 2016). Previous studies have found that DAS28‐CRP scores are negatively correlated with pain thresholds (Joharatnam et al. 2015) or positively correlated with arthritis‐specific pain scaling (Singh et al. 2020), confirming the relevance of this dataset to pain research. The presently analysed dataset was an 80 × 58 matrix (80 cases, 58 variables). For the present experiments, the preprocessed and split training/test versus validation data subsets from the previous publication were used (Rischke et al. 2023).

#### 3.4.2 Synthetic Dataset Demonstrating Structural Limits of Regression (‘FCPS::Atom’)

To illustrate structural limitations of regression analysis, we used the publicly available Atom dataset from the Fundamental Clustering and Projection Suite (FCPS) (Ultsch and Lötsch 2020). This artificial dataset comprises *n* = 800 points, evenly divided into two classes: ‘kernel’ and ‘hull.’ Kernel points are uniformly distributed within a sphere centered at the origin, while hull points are uniformly distributed on the surface of a larger concentric sphere (Figure S1). Thus, the dataset represents a three‐dimensional, two‐class problem stored in a numerical matrix of size 800 × 3.

## 4 Results

Results are reported for each dataset, which enhances emphasis on its particularities and solutions for unequivocal and comprehensive identification of trait‐relevant features.

### 4.1 Pain‐Thresholds Quantitative Sensory Testing Dataset (‘pain_thresholds_sex’)

#### 4.1.1 Machine Learning‐Based Feature Interpretation

The motivating example showed that, in some scenarios, such as LASSO feature selection, the unselected variables still contained sufficient information to enable certain machine learning algorithms to assign sex to new individuals. From the successful Phase 0 results (Table 1), the smallest feature set achieving classification success was Pressure2, Pressure and the pain threshold to punctate mechanical stimuli after sensitisation with topical capsaicin cream, as selected by Boruta (Figure 1A,C). This outcome aligned with the previously published finding that mechanical pain, particularly blunt pressure pain, plays the dominant role in sex classification (Lötsch et al. 2023). It is worth noting that pain thresholds to 5‐Hz sine wave electrical stimuli were ranked second in importance (Figure 1B), although the Boruta algorithm ultimately did not identify them as ‘confirmed’ significant. However, LASSO identified electrical stimuli and returned them as selected. Additionally, LASSO selected the menthol sensitisation effect on cold pain perception as relevant, suggesting a minor contribution from other variables, as reflected in variable importance magnitudes (Figure 1B,C).

Subsequent phases resolved this uncertainty. Phase 1's backward elimination further reduced the feature set to Pressure2. In Phase 2, each rejected feature was individually tested for independent predictive ability, and none demonstrated successful classification, confirming their exclusion. Phase 3 verified that the selected features consistently enabled classification, while the rejected features, when tested as a group, failed to allow any classifier to perform robustly (95% confidence interval above 0.5) on new cases. This confirmed that the final selected features (Pressure2, Pressure and electrical current; Table S1) were necessary and sufficient for reliable sex classification in new cases, while the remaining unselected features did not contribute independently or collectively to classification performance (Table 1).

Thus, interpretation of the quantitative sensory testing data suggests that, as reported previously (Lötsch et al. 2023), mechanical pain produced by blunt pressure stimuli plays the dominant role. An additional feature was electrical pain. Its maintenance in the final feature set reflects its ranking in the Boruta variance importance measure (Figure 1B).

#### 4.1.2 Identification of Relevant Pain Measures Using Classical Statistics

**FIGURE 3:**
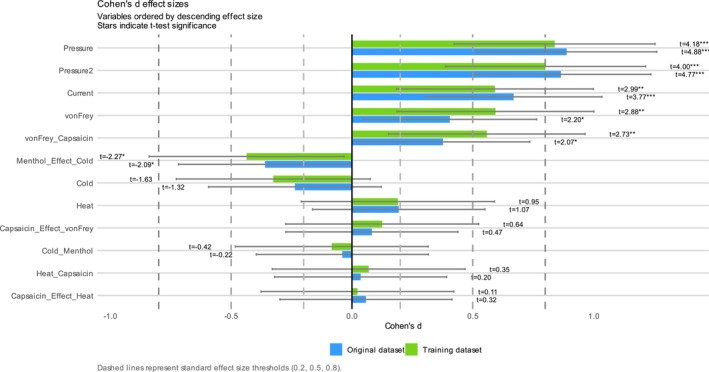
Pain thresholds dataset: Effect size analysis using Cohen's *d* with 95% Bootstrap confidence intervals comparing two groups in the pain thresholds dataset. Bars represent Cohen's *d* values for each variable, quantifying the standardised mean difference between groups. Error bars indicate 95% confidence intervals. Associated *t*‐test statistics and significance levels are annotated directly on the plot, with asterisks denoting significance thresholds (*p < 0.05, **p < 0.01, ***p < 0.001). Vertical dashed lines mark conventional benchmarks for small (0.2), medium (0.5) and large (0.8) effect sizes, facilitating interpretation of the magnitude of group differences. The original complete dataset and the 80% training data subset are compared.

Splitting the dataset into 80% for training and 20% for validation, which is common in machine learning but uncommon in classical statistics, led to minor changes in the observed effect sizes in the training subset. All diagnostics were derived from machine learning rather than the full dataset used for statistical analyses (e.g., Cohen's *d* and *t*‐test results). However, all effect directions and levels of statistical significance were preserved (Figure 3).

**FIGURE 4:**
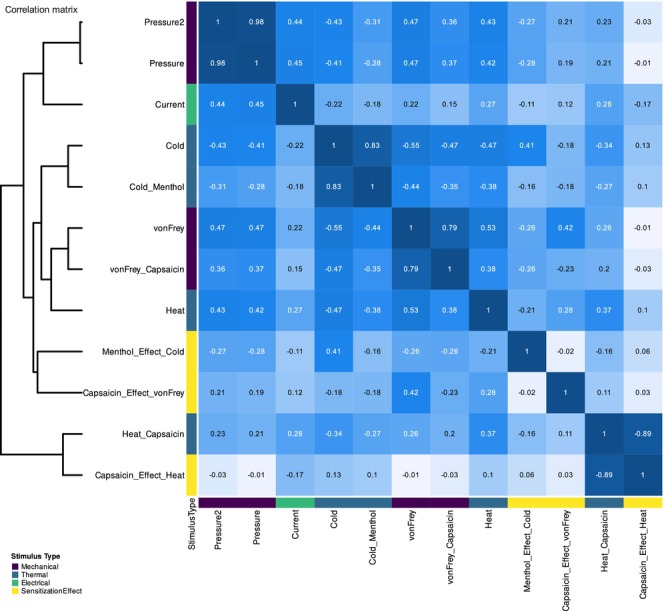
Pain thresholds dataset: Heat map of absolute Pearson correlation coefficients among pain threshold variables. The matrix visualises pairwise correlations, with hierarchical clustering (Ward's linkage; Ward 1963) applied to both rows and columns to visually enhance groups of related variables. Colour intensity indicates correlation magnitude according to the colour scale, ranging from low (light) to high (dark) correlations. Numeric correlation values are displayed within each cell. Annotations alongside rows and columns indicate stimulus type categories as coloured bars, and a corresponding legend identifies these groups.

The correlation matrix revealed strong correlations among variables, particularly between the two intentionally tailored pressure pain features (Figure 4). In addition, the sensitisation procedures produced highly correlated variables when considered separately or when expressed as differences between conditions with and without sensitisation. This outcome was expected. However, there was no prior formulation of research questions regarding the relevance of individual thresholds or sensitisation effects to the task of exploring sex differences in pain sensitivity.

##### 4.1.2.1 Apparent Failure of Feature Identification in the Complete Dataset

In the full modified dataset (with the pressure pain threshold duplicated and noise added), binary logistic regression initially suggested that pressure pain thresholds were not significant predictors (Table 2), despite consistent prior evidence to the contrary (see effect sizes below and previous analysis in Lötsch et al. 2023). In this analysis, only electrical pain thresholds reached statistical significance. Removing the duplicated pressure threshold variable restored the predictive role of the original pressure pain thresholds, but the regression output also contained errors and non‐numeric results (NA or NaN = ‘not a number’), caused by perfect multicollinear variables in the dataset. Both R and SPSS (Version 27 for Linux, IBM Corp., Armonk, NY, USA) produced similar results (see https://github.com/JornLotsch/pain‐threshold‐analysis/blob/master/Pheno_125_SPSS_regression_complete_dataset.pdf). Each issued warnings about perfect multicollinearity and handled these variables before proceeding but offered no explicit diagnostic values about high multicollinearity like the duplicated pressure threshold variable. R excluded these variables from the analysis, while SPSS reduced the degrees of freedom for these variables.

**TABLE 2:** Pain thresholds dataset: Logistic regression results, shown both with collinearity unaddressed (original and modified version by copying the pain pressure threshold) and after collinearity was explicitly addressed (either by removing variables with high variance inflation factors or by retaining only those variables).

	Variables	Original pain dataset					Modified pain dataset				
		Estimate	Std. error	*z* value	Pr(>|*z*|)	Significance	Estimate	Std. error	*z* value	Pr(>|*z*|)	Significance
Collinearity unaddressed	(Intercept)	−0.2741	0.2065	−1.327	0.18435	—	−0.2706	0.2067	−1.309	0.1905	—
	Heat	0.2339	0.2573	0.909	0.36334	—	0.2346	0.2575	0.911	0.3622	—
	Pressure	−0.8264	0.2766	−2.988	0.00281	**	−0.4554	1.2899	−0.353	0.7241	—
	Current	−0.5239	0.2439	−2.148	0.03172	*	−0.5264	0.2447	−2.151	0.0314	*
	Heat_Capsaicin	0.2435	0.2365	1.03	0.30322	—	0.2492	0.2383	1.046	0.2957	—
	Capsaicin_Effect_Heat	NA	NA	NA	NA	—	NA	NA	NA	NA	—
	Cold	0.3347	0.4538	0.738	0.4608	—	0.327	0.4547	0.719	0.472	—
	Cold_Menthol	−0.5164	0.4056	−1.273	0.20291	—	−0.517	0.4064	−1.272	0.2034	—
	Menthol_Effect_Cold	NA	NA	NA	NA	—	NA	NA	NA	NA	—
	vonFrey	−0.1284	0.3701	−0.347	0.72869	—	−0.1239	0.3715	−0.333	0.7388	—
	vonFrey_Capsaicin	−0.163	0.3359	−0.485	0.62744	—	−0.1696	0.3373	−0.503	0.615	—
	Capsaicin_Effect_vonFrey	NA	NA	NA	NA	—	NA	NA	NA	NA	—
	Pressure2					—	−0.3948	1.3471	−0.293	0.7694	—

	Variable	Pain dataset; VIF removed					Pain dataset; only VIF				
		Estimate	Std. error	*z* value	Pr(>|*z*|)	Significance	Estimate	Std. error	*z* value	Pr(>|*z*|)	Significance
Collinearity addressed	(Intercept)	−0.2741	0.2065	−1.327	0.18435	—	−0.23905	0.19817	−1.206	0.227701	—
	Heat	0.2339	0.2573	0.909	0.36334	—					—
	Pressure	−0.8264	0.2766	−2.988	0.00281	**					—
	Current	−0.5239	0.2439	−2.148	0.03172	*					—
	Heat_Capsaicin	0.2435	0.2365	1.03	0.30322	—					—
	Capsaicin_Effect_Heat					—	−0.12392	0.20349	−0.609	0.542547	—
	Cold	0.3347	0.4538	0.738	0.4608	—					—
	Cold_Menthol	−0.5164	0.4056	−1.273	0.20291	—					—
	Menthol_Effect_Cold					—	0.2091	0.22207	0.942	0.346384	—
	vonFrey	−0.1284	0.3701	−0.347	0.72869	—					—
	vonFrey_Capsaicin	−0.163	0.3359	−0.485	0.62744	—					—
	Capsaicin_Effect_vonFrey					—	0.07974	0.20264	0.394	0.693937	—
	Pressure2					—	−0.97119	0.2526	−3.845	0.000121	***

*Note:* For each model, regression coefficients (Estimate), standard errors, test statistics (*z* value) and corresponding *p*‐values (Pr(>|*z*|)) are reported, together with conventional significance markers (*p < 0.05; **p < 0.01; ***p < 0.001; — not significant).

##### 4.1.2.2 Necessity of Deliberate Variable Removal to Resolve Multicollinearity

**FIGURE 5:**
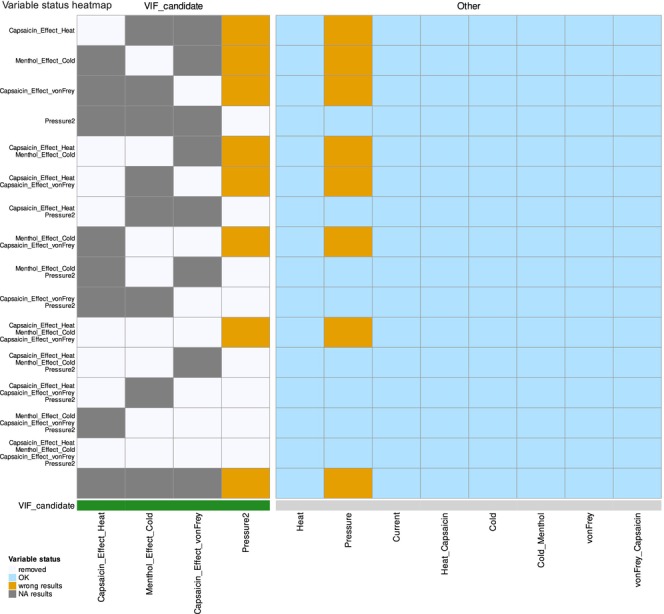
Pain thresholds dataset: Heat map summarising the status of all variables across multiple logistic regression model subsets. Each row represents a distinct model in which specific variable(s), indicated by the row name, were removed prior to analysis; columns correspond to all variables in the dataset. The cell colour codes reflect variable status within each model: Grey (‘removed’), indicating that the variable was excluded from the regression for that model run; light blue (‘OK’), where the regression analysis was successfully performed with this variable included and the statistical results agreed with the consistent findings throughout the analyses of this dataset; dark yellow (‘wrong results’), indicating that the variable was included but the regression produced invalid results that contrasted with the correct results according to the consistent analysis of this dataset; and dark grey (‘NA or aliased/collinear’), indicating that the variable could not be evaluated due to collinearity, aliasing or the regression produced missing results (NA) for this variable. Columns are marked to indicate whether each variable was a candidate for exclusion due to a high variance inflation factor (VIF > 10). The bottom row corresponds to the full model with no variables removed, serving as a reference run.

Variance inflation factor analysis confirmed multicollinearity but failed until removal of aliased variables, defined as perfectly collinear sensitisation‐effect differences. Excluding such collinear variables or alternatively retaining only one representative variable yielded error‐free regression results, which consistently identified pressure pain thresholds as the key predictors for sex classification from quantitative sensory testing (Table 2). However, stepwise removal of variables suspected of introducing collinearity (the duplicated pressure threshold and the three sensitisation measures) resulted in variable regression outcomes, sometimes with ‘NA’ values due to colinear or aliased variables (Figure 5). Some combinations even produced misleading inferences about the role of pressure pain thresholds, that is, the respective variables lacked significance despite consistent evidence that they were the driving variables in this dataset for the sex segregation.

##### 4.1.2.3 Penalised Regression as an Alternative to Resolve Multicollinearity

Penalised regression resolved the multicollinearity issues encountered with classical logistic regression; however, it introduced new uncertainty because its three implementations (ridge, lasso, elastic net) did not produce identical results (Table S2). The three final features identified in the preceding machine learning framework and thus shown to suit predictive tasks for identifying sex from quantitative sensory testing results were recovered by ridge and elastic net (all three) or partially by lasso (the two original variables but not the duplicated pressure threshold). While lasso identified no further variables, the other two variants selected additional features that had appeared in the original feature selection but failed to enable any algorithms to classify new cases better than chance level.

### 4.2 Psoriatic Arthritis Dataset (‘PsA DAS28‐CRP’)

#### 4.2.1 Machine Learning‐Based Feature Interpretation

**FIGURE 6:**
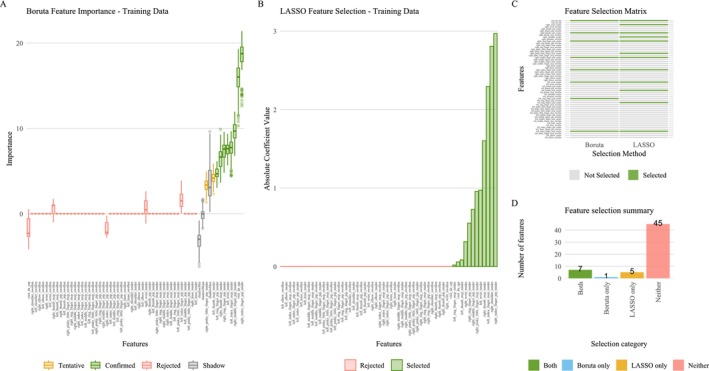
PsA DAS28‐CRP dataset: Feature selection results using Boruta and LASSO methods. The first two panels show Boruta importance scores as boxplots and LASSO absolute coefficient values as barplots, with features coloured by selection status (‘Confirmed’ or ‘Selected’ in green, ‘Tentative’ in orange and ‘Rejected’ in salmon). The third panel presents a heat map matrix illustrating feature selection overlap between Boruta and LASSO, with green tiles indicating selection by each method. The fourth panel summarises the counts of features uniquely selected by each method, selected by both methods or rejected by both.

In the psoriatic arthritis dataset, both Boruta and LASSO highlighted the patient's self‐rated global health assessment, a core DAS28‐CRP component, together with tenderness in several peripheral finger joints (Figure 6). Among these, tenderness of the right index finger was again selected, consistent with previous work identifying the right index MCP joint as the most informative single DAS28‐CRP component for staging psoriatic arthritis activity (Rischke et al. 2023). While Boruta yielded a more parsimonious predictor set and LASSO retained additional joints, both approaches converged global health assessment and localised finger joint tenderness as key contributors.

Applying the full four‐phase feature selection framework ultimately identified four DAS28‐CRP items as sufficient for reliable classification of active versus inactive PsA: right_index_finger_pip_tender, visit_da_sga, right_ring_finger_pip_tender and right_middle_finger_pip_tender (Table S1). Classification remained successful when restricted to these selected features, and further execution resulted in classification success with the then unselected features and was therefore dismissed, whereas performance dropped to chance level when only the unselected features were used, indicating no robust classification success from the remaining variables (Table 3).

**TABLE 3:** PsA DAS28‐CRP dataset: Classification performance based on 100 repeated runs. Feature selection pipeline results.

Dataset	Features	RF_BA	LR_BA	KNN_BA	C50_BA	SVM_BA	Classification_Success	Phase
All_Features	58	0.500 [0.500, 0.750]	0.750 [0.625, 0.875]	0.500 [0.500, 0.500]	0.750 [0.650, 0.801]	0.950 [0.900, 1.000]	1	Phase_0_Full
Boruta_Selected	8	0.750 [0.619, 0.875]	1.000 [0.700, 1.000]	0.500 [0.500, 0.625]	0.750 [0.700, 0.801]	0.950 [0.950, 1.000]	1	Phase_0_Full
Boruta_Rejected	50	0.500 [0.474, 0.500]	0.575 [0.450, 0.750]	0.500 [0.500, 0.500]	0.500 [0.450, 0.500]	0.575 [0.500, 0.750]	0	Phase_0_Full
LASSO_Selected	9	0.750 [0.750, 0.875]	0.750 [0.500, 0.875]	0.500 [0.500, 0.500]	0.750 [0.700, 0.801]	0.900 [0.900, 1.000]	1	Phase_0_Full
LASSO_Rejected	49	0.500 [0.500, 0.500]	0.625 [0.500, 0.750]	0.500 [0.500, 0.500]	0.500 [0.450, 0.500]	0.575 [0.450, 0.750]	0	Phase_0_Full
Boruta_LASSO_Selected	10	0.750 [0.619, 0.875]	0.750 [0.500, 0.875]	0.500 [0.500, 0.500]	0.750 [0.700, 0.801]	0.900 [0.900, 1.000]	1	Phase_0_Full
Boruta_LASSO_Rejected	48	0.500 [0.500, 0.500]	0.625 [0.450, 0.750]	0.500 [0.500, 0.500]	0.500 [0.450, 0.500]	0.575 [0.500, 0.750]	0	Phase_0_Full
All_Features	4	1.000 [0.849, 1.000]	0.950 [0.825, 1.000]	0.825 [0.625, 1.000]	0.875 [0.750, 0.875]	1.000 [0.950, 1.000]	1	Phase_3_Final_Selected
Boruta_Selected	4	1.000 [0.849, 1.000]	0.950 [0.825, 1.000]	0.825 [0.625, 1.000]	0.875 [0.750, 0.875]	1.000 [0.950, 1.000]	1	Phase_3_Final_Selected
LASSO_Selected	4	1.000 [0.849, 1.000]	0.950 [0.825, 1.000]	0.825 [0.625, 1.000]	0.875 [0.750, 0.875]	1.000 [0.950, 1.000]	1	Phase_3_Final_Selected
Boruta_LASSO_Selected	4	1.000 [0.849, 1.000]	0.950 [0.825, 1.000]	0.825 [0.625, 1.000]	0.875 [0.750, 0.875]	1.000 [0.950, 1.000]	1	Phase_3_Final_Selected
All_Features	54	0.500 [0.500, 0.500]	0.625 [0.474, 0.750]	0.500 [0.500, 0.500]	0.500 [0.400, 0.500]	0.575 [0.500, 0.700]	0	Phase_3_Final_Rejected
Boruta_Selected	6	0.500 [0.450, 0.500]	0.450 [0.400, 0.500]	0.500 [0.500, 0.500]	0.500 [0.450, 0.500]	0.400 [0.400, 0.500]	0	Phase_3_Final_Rejected
Boruta_Rejected	48	0.500 [0.500, 0.500]	0.575 [0.450, 0.750]	0.500 [0.500, 0.500]	0.500 [0.450, 0.500]	0.575 [0.474, 0.750]	0	Phase_3_Final_Rejected
LASSO_Selected	5	0.500 [0.450, 0.500]	0.500 [0.450, 0.500]	0.500 [0.500, 0.500]	0.500 [0.450, 0.500]	0.450 [0.450, 0.500]	0	Phase_3_Final_Rejected
LASSO_Rejected	49	0.500 [0.474, 0.500]	0.575 [0.450, 0.726]	0.500 [0.500, 0.500]	0.500 [0.450, 0.500]	0.625 [0.500, 0.750]	0	Phase_3_Final_Rejected
Boruta_LASSO_Selected	6	0.500 [0.450, 0.500]	0.450 [0.400, 0.500]	0.500 [0.500, 0.500]	0.500 [0.450, 0.500]	0.400 [0.400, 0.500]	0	Phase_3_Final_Rejected
Boruta_LASSO_Rejected	48	0.500 [0.500, 0.500]	0.575 [0.450, 0.750]	0.500 [0.500, 0.500]	0.500 [0.450, 0.500]	0.575 [0.474, 0.750]	0	Phase_3_Final_Rejected

*Note:* Median balanced accuracy (BA) with non‐parametric 95% confidence intervals from 100 bootstrap iterations using five classifiers: Random Forest (RF), Logistic Regression (LR), K‐nearest neighbours (KNN), C5.0 decision trees (C50) and support vector machines (SVM). Classification_Success indicates whether any classifier achieved a lower confidence interval boundary exceeding 0.5 (1 = success, 0 = failure). Phase_0_Full: Initial screening of all feature combinations from the complete 58‐feature dataset, including the full set and subsets identified by Boruta and LASSO feature selection methods, along with their unions, intersections and rejected features. Phase_3_Final_Selected: The minimal feature set (right_index_finger_pip_tender, visit_da_sga, right_ring_finger_pip_tender, right_middle_finger_pip_tender) after backward elimination and rescue procedures, tested to verify it cannot be further reduced while maintaining classification success. Phase_3_Final_Rejected: The 54 excluded features tested individually and in all combinations resulting from the various feature selection methods; none achieved classification success, confirming their exclusion (Names of the respective features omitted due to their large number exceeding space).

#### 4.2.2 Statistics‐Based Feature Interpretation

##### 4.2.2.1 Lack of Identification of Relevant Pain Measures Using Classical Statistics

Logistic regression applied to the complete unsplit psoriatic arthritis dataset failed to identify significant predictors, irrespective of whether collinear variables were retained or removed (Table S3). In the full model with 58 variables, 36 coefficients were undefined due to perfect multicollinearity, and all remaining estimates were non‐significant (*p* ≈ 0.999). Reducing the model to 22 features by excluding collinear variables did not resolve this issue, as the regression again produced only non‐significant coefficients. These results indicate that, in this dataset, regression was unable to extract meaningful predictors and is structurally unsuitable for classification. For comparison, SPSS led to the same results (see https://github.com/JornLotsch/pain‐threshold‐analysis/blob/master/PSA_das28crp_SPSS_regression_complete_dataset.pdf). These difficulties are partly attributable to the structure of the dataset, where most predictors are binary (0/1, joint affected no/yes, scores). While such variables are in principle suitable for logistic regression, the high degree of redundancy among them combined with the small sample size produced severe multicollinearity and model instability.

##### 4.2.2.2 Penalised Regression Results in the PsA Dataset

Unlike classical logistic regression, penalised approaches (ridge, lasso, elastic net) produced defined coefficients; however, they introduced new uncertainty through inconsistent variable selection across methods (Table S2). The four final features identified by the machine learning framework, that is, right_index_finger_pip_tender, visit_da_sga, right_ring_finger_pip_tender and right_middle_finger_pip_tender, were recovered by all three methods alongside numerous additional variables. While lasso and elastic net selected some further features, ridge regression retained nearly all variables with coefficients exceeding 0.05 (excluding only zero‐variance features), an implausible result given that such extensive feature sets failed to enable classification beyond chance level in the machine learning analysis (Table 3).

### 4.3 Regression Failure in a Non‐Linearly Separable Synthetic Dataset

Limits of the regression model were demonstrated using the ‘Atom’ synthetic dataset from the FCPS collection. Although the two classes are visually distinct (Figure S1), they are not linearly separable, so no single linear boundary can perfectly distinguish them. Classical logistic regression applied to the full dataset confirmed these limitations: none of the predictor coefficients reached statistical significance (*p* = 0.15–0.46; Table 4), reflecting the model's poor performance in capturing the underlying structure.

**TABLE 4:** Results for the FCPS::Atom dataset.

**Classical logistic regression**						
**Variables**	**Estimate**	**Std. error**	***z* value**	**Pr(>|*z*|)**	**Significance**	
X1	0.004362	0.003792	1.15	0.25	—	
X2	0.005513	0.003859	1.429	0.153	—	
X3	−0.0022	0.002967	−0.741	0.458	—	
**Penalised logistic regression**						
**Variable**	**ridge_coef**	**ridge_selected**	**lasso_coef**	**lasso_selected**	**elastic_coef**	**elastic_selected**
X1	6.34E‐05	FALSE	0	FALSE	0.00341	TRUE
X2	7.75E‐05	FALSE	0	FALSE	0.004469	TRUE
X3	−3.08E‐05	FALSE	0	FALSE	−0.00145	TRUE
**Machine learning: Balanced accuracy in validation**						
***y***	**RF_BA**	**LR_BA**	**pLR_BA**	**KNN_BA**	**C50_BA**	**SVM_BA**
All features	0.992 [0.97; 1]	0.631 [0.555; 0.687]	0.628 [0.546; 0.683]	1 [1; 1]	1 [1; 1]	1 [1; 1]

*Note:* The upper panel reports coefficients from classical logistic regression (Estimate), their standard errors, Wald test statistics (*z* value) and corresponding *p‐*values (Pr(>|*z*|)), together with conventional significance markers indicating predictors associated with group membership. The middle panel shows coefficients from ridge, lasso, and elastic net penalised logistic regression (ridge_coef, lasso_coef, elastic_coef), and corresponding selection indicators (ridge_selected, lasso_selected, elastic_selected), where variables are marked as selected if their penalised coefficient is non‐zero (for lasso and elastic net) or exceeds a predefined absolute threshold (for ridge). The lower panel presents mean balanced accuracy (BA) with 95% confidence intervals in the validation data for six classifiers—random forest (RF), logistic regression (LR), penalised logistic regression (pLR), k‐nearest neighbour (KNN), C5.0 decision trees (C50) and support vector machine (SVM)—applied to the full feature set.

Penalised regression offered only a partial remedy. Among the variants tested, only elastic net identified the three relevant variables of the 3‐dimensional artificial dataset (in agreement with Boruta, which identified all its three variables as ‘confirmed’ significant), while ridge and lasso did not. However, even elastic net failed to improve classification performance beyond the classical logistic regression model (Table 4).

Balanced accuracy comparisons further illustrated this limitation (Table 4). Logistic regression achieved only 0.63 (95% CI: 0.56–0.69), indicating discrimination only slightly above chance. In contrast, nonlinear machine learning models performed substantially better: random forests reached a balanced accuracy of 0.98 (95% CI: 0.96–0.99), and both k‐nearest neighbours and the decision tree (C5.0) achieved perfect separation with median balanced accuracy values of 1.0 in this simple dataset. Similar findings across other FCPS datasets, and prior reports, confirm the general limitations of regression models and the superior capacity of machine learning methods to detect structure in non‐linearly separable data (Lötsch and Ultsch 2023a).

## 5 Discussion

Machine learning‐based feature selection offers advantages over traditional *p*‐value‐based approaches for identifying variables relevant to a trait but also introduces uncertainty about which features truly drive the observed differences. It assumes that variables enabling accurate classification of new cases (e.g., male/female, healthy/diseased) define the class structure, and biological interpretation often centres on these selected features, as it does on statistically significant variables in classical analyses. However, this assumption becomes problematic when excluded variables still permit accurate classification, indicating that biologically relevant information may extend beyond the selected subset (Bontonou et al. 2025; Lötsch and Ultsch 2022).

To address this ambiguity, we developed a framework that combines iterative evaluation with a mixture‐of‐experts approach integrating multiple algorithms and domain knowledge to offset method‐specific limitations (details below). This framework helps identify trait‐relevant variables more comprehensively than classical methods through (i) iterative evaluation of each variable's contribution to model training and (ii) joint assessment by algorithmic and human experts to confirm or refute candidate features.

### 5.1 Framework Components: Iterative Evaluation and Mixture of Experts

#### 5.1.1 Iterative Evaluation of Variables

Iterative evaluation of unselected variables reduces uncertainty by testing their ability to improve classification accuracy. As implemented (Figure 2), this strategy assesses individual feature contributions both independently and combined with other variables, ensuring important variables are not prematurely excluded (Lötsch and Ultsch 2022). This complements traditional methods focused on minimal informative subsets by emphasising refined relevance assessment and enabling deeper insights into data‐driven patterns (Lötsch and Ultsch 2023b).

#### 5.1.2 Computational Expertise: Mixture of Algorithms

The Mixture of Experts (MoE) principle provides the computational foundation. MoE combines multiple feature selection and classification algorithms, leveraging their complementary strengths in complex biomedical datasets (Hu et al. 1997; Khadirnaikar et al. 2023). Feature selection employed LASSO and random forests; classification used random forests, logistic regression, k‐nearest neighbours, C5.0 and support vector machines. However, classifier success varied: interpretable models like C5.0 failed for the PsA dataset, while random forests, though powerful, did not always succeed (Tables 1 and 3). These results underscore the value of algorithmic diversity for addressing dataset‐specific complexities (Hu et al. 1997; Khadirnaikar et al. 2023; Khalili 2010; Lötsch and Ultsch 2023a; Pradier et al. 2021).

#### 5.1.3 Biomedical Expertise: Human Domain Expert Interpretation

Human biomedical expertise constitutes an equally critical MoE component. While machine learning excels at computational efficiency and pattern recognition, it may obscure true biological drivers. Expert interpretation bridges this gap, providing contextual insights that enhance interpretability and biological accuracy. Given the ambiguous machine learning outcomes (cold sensitisation relevance for sex differences) and counterintuitive statistical failures (PsA activity could not be deduced from its constituent variables), domain expertise becomes essential for interpreting pain‐related datasets.

##### 5.1.3.1 Application to Pain and Disease Datasets

###### 5.1.3.1.1 Sex Differences in Experimental Pain Thresholds

Sex differences in experimental pain thresholds were confirmed using the new feature selection framework. Applied to the same dataset but with different coding and a focus on any sufficient classifier rather than minimal measures, the framework reproduced the dominant role of blunt pressure pain thresholds in sex differences in pain perception (Lötsch et al. 2023). Strong sex effects for mechanical pain align with biological mechanisms, including differential Piezo2 expression (Shin et al. 2021), acid‐sensing ion channels and hormonal modulation of nociceptor sensitivity (Mogil 2012; Sorge and Totsch 2017).

The framework refined this further, pinpointing C‐fibre mediated pain. It identified 5‐Hz sine‐wave electrical pain thresholds (elicited via Neurometer CPT constant current stimulator) as the second key feature enabling sex classification by trained algorithms. This aligns with parallel nociceptive pathways (Basbaum et al. 2009) and evidence that both blunt pressure (Cline et al. 1989; Culp et al. 1989; Kilo et al. 1994; Koltzenburg et al. 1992) and 5‐Hz stimulation (Kiso et al. 2001; Masson et al. 1989; Pitei et al. 1994; Veves et al. 1994; Wallace et al. 1996) predominantly activate C‐fibres. Thus, the framework highlighted physiologically relevant QST measures for sex differences, providing a literature‐supported characterisation. These results for blunt pressure and electrical (5‐Hz sine waves) pain also align with previous findings that effects of remifentanil were mainly seen in these two stimuli in a human volunteer pharmacological study (Lötsch and Angst 2003).

Although prior analyses largely excluded thermal pain (Lötsch et al. 2023), alternative selectors like LASSO suggested cold pain sensitisation effects, which Boruta ignored. This method dependence emphasises mixture‐of‐experts approaches and validation. LASSO's signals merit follow‐up given context‐dependent thermal sex differences (females more sensitive to heat/cold) (Goreis et al. 2025; Gulati et al. 2023; Mogil 2020; Osborne and Davis 2022), modulated by protocols and hormones (Keogh 2022; Keogh and Boerner 2024), with moderate effect sizes (Cohen's *d* ≈ 0.5–0.6) (Liossi et al. 2024; Robinson et al. 1998). Menthol sensitivity shows no clear human sex differences, with evidence mostly from rodents (Alarcón‐Alarcón et al. 2022; Caudle et al. 2017; Kondrats'kyĭ et al. 2009).

###### 5.1.3.1.2 Clinical Markers of Psoriatic Arthritis Activity

The PsA dataset analysis was straightforward given that the target DAS28‐CRP score is calculated from components based on established clinical knowledge: small finger joints are often earliest and most affected in PsA and rheumatoid arthritis (Mease 2011; Singh et al. 2016). Finger joint tenderness marks localised synovial inflammation and joint damage correlating with systemic immune activity via IL‐17/IL‐23 pathways (Sorge and Totsch 2017). Right index finger tenderness as a key signal for staging PsA activity is biologically explicable through site‐specific mechanical loading (dominant hand use) interacting with localised immune activation (Vasconcelos et al. 2022). However, the small PsA cohort and absent DAS28‐CRP components (several variables always zero; see figure 2 in Rischke et al. (2023)) mean this interpretation, while clinically reasonable, is not derived from balanced component analysis.

### 5.2 Limitations of Classical Statistical Approaches and Possible Remedies

Classical statistical methods struggle with the highly structured data scenarios encountered here, revealing fundamental limitations when reverting from machine learning frameworks.

#### 5.2.1 Challenges With Highly Correlated Variables

Regression analysis has fundamental limitations with highly correlated variables (Lötsch et al. 2022; Rügamer et al. 2024), as expected, particularly evident in the engineered pain threshold dataset. While extreme correlations (*r* > 0.95) are rare and should prompt checking for errors (Martina Udovicic 2007), they cannot be excluded in practice. Real‐world biomedical datasets exhibit high correlations: plasma and urine dextromethorphan levels (*r* = 0.934) (Lötsch et al. 2009), MCP‐1 change versus peak pain ratings (*r* = 0.85) (Cruz‐Almeida et al. 2012), DNA methylation markers in persistent pain (*r* = 0.84) (Kringel et al. 2019), heat pain thresholds and capsaicin sensitisation (|*r*| = 0.84; Figure 4), and hepatic arterial blood flow (*r* = 0.98) (Chen et al. 1991). Genetic markers in high linkage disequilibrium provide another example of measurement overlap.

Standard practices exclude variables above certain thresholds (e.g., Spearman's coefficients > 0.90; Li et al. 2024) or use computational strategies like the ‘findCorrelation’ function (Kuhn 2018). However, such exclusions introduce premature decisions about relevance and complicate interpretation. When removed variables pertain to pathways relevant to the study focus, exclusion could obscure insight. Classical logistic regression struggled with these structures and required variable exclusion to prevent analytical errors. Our framework avoids these pitfalls and ensures that no relevant variables are prematurely dismissed.

#### 5.2.2 Structural Limitations of Regression

Regression techniques show intrinsic limitations which is analogous to early neural network models that lacked hidden layers and could only represent simple relationships. Just as those early models could not solve certain classification problems, standard regression approaches may fail when relationships between variables are more complex. As Minsky and Papert (Minsky and Papert 1969) demonstrated, the perceptron (Rosenblatt 1958) could not solve linearly non‐separable problems without hidden layers, constraints later overcome by more flexible architectures (Takefuji 2025).

In the engineered pain‐threshold dataset with nearly identical variables, the modest performance of ordinary logistic regression was therefore expected. More surprising was its insufficient reconstruction of PsA activity in the unmodified real‐world dataset. While this outcome can partly be explained by the limited cohort size and zero‐valued variables (Rischke et al. 2023), it highlights that purely linear models may struggle with overlapping or interacting predictors. Further methods such as Firth‐corrected logistic regression (Firth 1993) can alleviate certain technical issues but do not address deeper structural challenges.

Penalised regression provided partial improvement, effectively identifying the relevant variables in the datasets and, to some extent, rehabilitating regression as a modelling tool. However, this came at the cost of losing the intuitive *p*‐value framework for variable selection, and different penalty implementations produced varying feature sets. This was particularly evident in the artificial dataset, where only elastic net, but neither ridge nor LASSO identified all three relevant variables. Moreover, elastic‐net regression performed about as poorly as classical logistic regression when used as a pure classifier, in contrast to near‐perfect results from tree‐based learners. These findings suggest that the linear formulation of penalised logistic regression limits its ability to capture non‐linear or locally complex patterns, even when the relevant variables are correctly identified.

Overall, regression methods remain valuable for their interpretability, robustness and familiarity, yet they face challenges in datasets characterised by non‐linear, collinear or overlapping structures. The observed limitations support a complementary perspective, where regression is integrated with machine learning or expert‐guided frameworks to better capture the complexity of biomedical data.

### 5.3 Practical Considerations

#### 5.3.1 Advantages of Machine Learning‐Based Feature Selection

Our framework addresses interpretive ambiguities through iterative validation and a mixture‐of‐experts strategy. Advantages include the handling of complex variable structures that is challenging classical regression (highly correlated features, non‐linearly separable data), providing robustness through diverse algorithms when individual methods fail or disagree, and ensuring potentially relevant variables are not prematurely excluded via iterative rescue mechanisms. The classification‐based success criterion directly addresses whether features reliably distinguish groups in new cases, emphasising predictive validity aligned with clinical and translational goals where generalisability matters more than parameter estimation.

#### 5.3.2 Disadvantages and Challenges of Machine Learning Approaches

##### 5.3.2.1 Standardisation Issues

Unlike *p*‐values, which have widely accepted interpretation thresholds, measures of classification performance do not have universal benchmarks. As a result, interpreting whether a given level of accuracy is meaningful often depends on context, further motivating the use of comparative and iterative evaluation strategies. While balanced accuracy has scientific support, it remains a methodological choice rather than an established standard. Classification performance varies across algorithms, implementations and hyperparameters. Our results differed numerically from earlier Python‐based analyses (Lötsch et al. 2023; Rischke et al. 2023), though conclusions aligned, contrasting with typical statistical test reproducibility. Machine learning typically requires custom coding rather than validated point‐and‐click software, increasing susceptibility to implementation errors. Classification failure indicates only that no suitable algorithm was successfully implemented, not that features are definitively irrelevant. Code sharing, as implemented here, enables retrospective evaluation. As mentioned in the Section 3, coding for this report was facilitated by AI; however, the analyses were coded with AI, and definitely not by AI, meaning that coding skills will continue to be required, at least for the time being.

##### 5.3.2.2 Computational Demands

Machine learning substantially increases computational requirements. The pain threshold dataset required approximately 1 h on a 64‐core processor (up to 127 concurrent processes); the PsA dataset required 1.5 h. These timings illustrate the trade‐off: machine learning demands exceed classical regression (completing in seconds) by orders of magnitude, though by contemporary standards these demands remain modest.

#### 5.3.3 Interpretation and Reporting of Analytical Goals

##### 5.3.3.1 Defining Feature Relevance

Classical statistics considers variables passing significance thresholds as trait‐relevant. Machine learning defines relevance differently: selected features enable successful classifier training, but this relevance is not exclusive. Unselected variables may also contribute to group differences. Exclusive irrelevance can only be claimed if classification fails when restricted to unselected features, which is the objective of our framework.

Even with consistent framework signals, conclusions remain limited to tested algorithms and implementations. Conflicting signals may require additional analyses (e.g., variable importance rankings) and domain expertise, as illustrated when LASSO identified cold sensitisation effects that Boruta missed.

##### 5.3.3.2 Predictive Utility Versus Statistical Significance

This framework shifts focus from classical inference to prediction, aligning analytical choices with study objectives (Hohmann et al. 2017). While conventional statistics focuses on quantifying uncertainty around estimated effects, machine learning approaches prioritise the ability to make accurate predictions. These perspectives address different questions, and combining them can provide a more complete understanding of complex biological data (Shalev‐Shwartz and Ben‐David 2014). Accordingly, our analyses emphasise classification performance and variable relevance rather than *p‐*values.

Statistical significance and predictive utility capture different aspects of data behaviour. A variable may lack statistical significance yet still improve prediction accuracy (Lo et al. 2015), while a statistically significant effect may contribute little to classification. High *p‐*values often result from small sample size, noise or multicollinearity rather than a true absence of predictive information (Heston and King 2017).

This distinction was evident in our analyses. Regression frequently yielded non‐significant coefficients yet achieved moderate predictive accuracy, as seen in the PsA dataset. Penalisation slightly improved these results but did not fully resolve them, and depending on the implementation, additional variables without predictive relevance, as shown in our framework, were sometimes selected. A similar pattern appeared in the artificial dataset described earlier, where linear regression identified informative variables but could not represent the underlying class structure as effectively as nonlinear models. These findings illustrate that inference and prediction serve different scientific purposes, that is, understanding associations versus achieving accurate classification, and should be regarded as complementary rather than competing approaches in biomedical research.

#### 5.3.4 Method Plurality as Strategy

Neither classical statistics nor machine learning universally dominates. Regression failed on both datasets inferentially, encountering collinearity in engineered pain threshold data and unexpectedly failing to reconstruct PsA activity from its components. Machine learning succeeded where regression failed but introduced interpretive uncertainty requiring iterative validation and domain expertise. The optimal strategy may be method plurality: applying both paradigms, transparently reporting where they agree or diverge, and using biological and clinical expertise to resolve ambiguities. This extends the mixture‐of‐experts philosophy beyond individual algorithms to methodological frameworks themselves.

### 5.4 Limitations

The methods represent only a small subset of available algorithms (Lotsch and Ultsch 2017; Lötsch et al. 2022; Murphy 2012). This report used only two feature selection algorithms among many available (Guyon 2003). Earlier work combined 17 algorithms via item categorisation, potentially capturing a broader spectrum of feature relevance (Lötsch et al. 2024). The chosen classification algorithms covered major families for numerical tabular data, including tree‐based bagging (random forests), margin‐based hyperplane separation (SVM), distance‐based classification (kNN), and the interpretable C5.0 method particularly useful for pain‐related biomedical data (Lötsch and Malkusch 2021). Other classifier families, such as neural networks or naïve Bayes, are absent.

Inclusion of further methods would exceed this report's purpose of proposing a framework for machine learning‐based identification of trait‐relevant features while excluding likely non‐relevant features. Nonetheless, the framework is model‐agnostic; its core logic is independent of any specific algorithm, and additional feature selection methods or classifiers can be incorporated by future users according to their data and research questions.

## 6 Conclusions

This work introduces a unified framework for feature selection in pain research that integrates iterative evaluation with a mixture of experts principle into one cohesive approach improving interpretability of selected and unselected features. This extends previous methodological developments (Lötsch et al. 2023; Lötsch and Ultsch 2022, 2023b) which proposed the ‘reversibility’ of associations, finding the minimum informative feature set sufficient for classification and generally reconsidering initially unselected features. Unlike our earlier machine learning applications to pain classification (Lötsch et al. 2023; Lötsch and Ultsch 2022), which focused on predictive accuracy, the current framework systematically addresses both selected and unselected features: selected features are verified to enable classification individually or in combination, while unselected features are rigorously tested to confirm their inability to support classification. This dual validation strategy, presented here as a model‐agnostic framework, provides a systematic solution to the longstanding ambiguity between machine learning and statistical approaches regarding variable relevance in pain traits.

The framework addresses a key challenge: classifier success can persist even after removal of supposedly important features, revealing incomplete understanding rather than methodological failure. Through iterative evaluation across the full feature set applied to the same QST and PsA datasets analysed previously (Lötsch et al. 2023; Lötsch and Ultsch 2022), the framework now reveals not only which features matter most, but also guarantees that unselected features classify at chance level, providing a complete spectrum of feature relevance unavailable from earlier analyses. A principal insight concerns the continuum from classical inference through penalised regression to machine learning. Rather than viewing these as competing paradigms, the analysis highlights their complementary strengths: statistical models provide theoretical transparency, machine learning captures complex nonlinear relationships, and penalised regression bridges both domains by combining interpretability with regularisation. The framework demonstrates that machine learning can identify key variables driving pain traits as reliably as classical statistics, and in some cases substantially better, while simultaneously providing the interpretive clarity traditionally associated with statistical approaches. The mixture of experts principle extends beyond algorithmic diversity to include collaboration among computer science, machine learning and biomedical pain researchers. Such interdisciplinary integration is essential for translating computational outcomes into mechanistic understanding.

Overall, this framework delivers a generalisable, model‐agnostic (Box 1) methodology for biomedical studies that unites statistical rigour with predictive flexibility, providing sound, reliable and interpretable insights into complex pain‐related data.

## Author Contributions

J.L.: conceptualization, methodology, software, validation, formal analysis, investigation, resources, data curation, writing – original draft, writing – review and editing, visualization, supervision, project administration, funding acquisition. A.H.: methodology, writing – original draft, writing – review and editing. D.K.: validation, writing – original draft, writing – review and editing.

## Funding

J.L. was supported by the Deutsche Forschungsgemeinschaft (DFG LO 612/16‐1).

## Ethics Statement

The studies from which the biomedical datasets originate followed the Declaration of Helsinki and were approved by the Ethics Committee of Medical Faculty of the Goethe‐University, Frankfurt am Main, Germany (approval numbers 150/11 and 19‐492_5).

## Consent

All participants provided written informed consent.

## Conflicts of Interest

The authors declare no conflicts of interest.

## Supporting information


**Data S1:** ejp70221‐sup‐0001‐Supinfo.pdf.

## Data Availability

The biomedical data sets are available from the first author upon reasonable request. All code used for generating the present report is provided at https://github.com/JornLotsch/pain‐threshold‐analysis/tree/master, with the corresponding archived release accessible via Zenodo at https://doi.org/10.5281/zenodo.18256405. The Zenodo release provides the exact version of the code associated with the accepted paper, ensuring reproducibility of the published analyses.

## References

[ejp70221-bib-0001] Alarcón‐Alarcón, D. , D. Cabañero , J. de Andrés‐López , et al. 2022. “TRPM8 Contributes to Sex Dimorphism by Promoting Recovery of Normal Sensitivity in a Mouse Model of Chronic Migraine.” Nature Communications 13: 6304. 10.1038/s41467-022-33835-3 PMC9588003 36272975

[ejp70221-bib-0002] Basbaum, A. I. , D. M. Bautista , G. Scherrer , and D. Julius . 2009. “Cellular and Molecular Mechanisms of Pain.” Cell 139: 267–284. 19837031 10.1016/j.cell.2009.09.028 PMC2852643

[ejp70221-bib-0003] Bontonou, M. , A. Haget , M. Boulougouri , B. Audit , P. Borgnat , and J.‐M. Arbona . 2025. “A Comparative Analysis of Gene Expression Profiling by Statistical and Machine Learning Approaches.” Bioinformatics Advances 5: vbae199. 39897946 10.1093/bioadv/vbae199 PMC11783302

[ejp70221-bib-0004] Breiman, L. 2001. “Random Forests.” Machine Learning 45: 5–32.

[ejp70221-bib-0005] Brodersen, K. H. , C. S. Ong , K. E. Stephan , and J. M. Buhmann . 2010. “The Balanced Accuracy and Its Posterior Distribution.” In Pattern Recognition (ICPR), 2010 20th International Conference, 3121–3124. IEEE.

[ejp70221-bib-0006] Caudle, R. M. , S. L. Caudle , A. C. Jenkins , A. H. Ahn , and J. K. Neubert . 2017. “Sex Differences in Mouse Transient Receptor Potential Cation Channel, Subfamily M, Member 8 Expressing Trigeminal Ganglion Neurons.” PLoS One 12: e0176753. 28472061 10.1371/journal.pone.0176753 PMC5417611

[ejp70221-bib-0007] Chen, B. C. , S. C. Huang , G. Germano , et al. 1991. “Noninvasive Quantification of Hepatic Arterial Blood Flow With Nitrogen‐13‐Ammonia and Dynamic Positron Emission Tomography.” Journal of Nuclear Medicine 32: 2199–2206. 1744703

[ejp70221-bib-0008] Cline, M. A. , J. Ochoa , and H. E. Torebjork . 1989. “Chronic Hyperalgesia and Skin Warming Caused by Sensitized C Nociceptors.” Brain 112, no. Pt 3: 621–647. 2731024 10.1093/brain/112.3.621

[ejp70221-bib-0009] Cohen, J. 1992. “A Power Primer.” Psychological Bulletin 112: 155–159. 19565683 10.1037//0033-2909.112.1.155

[ejp70221-bib-0010] Cortes, C. , and V. Vapnik . 1995. “Support‐Vector Networks.” Machine Learning 20: 273–297.

[ejp70221-bib-0011] Cover, T. , and P. Hart . 1967. “Nearest Neighbor Pattern Classification.” IEEE Transactions on Information Theory 13: 21–27.

[ejp70221-bib-0012] Cruz‐Almeida, Y. , C. D. King , S. M. Wallet , and J. L. Riley 3rd . 2012. “Immune Biomarker Response Depends on Choice of Experimental Pain Stimulus in Healthy Adults: A Preliminary Study.” Pain Research and Treatment 2012: 538739. 23213513 10.1155/2012/538739 PMC3508574

[ejp70221-bib-0013] Culp, W. J. , J. Ochoa , M. Cline , and R. Dotson . 1989. “Heat and Mechanical Hyperalgesia Induced by Capsaicin. Cross Modality Threshold Modulation in Human C Nociceptors.” Brain 112: 1317–1331. 2804614 10.1093/brain/112.5.1317

[ejp70221-bib-0014] Doehring, A. , N. Küsener , K. Flühr , T. J. Neddermeyer , G. Schneider , and J. Lötsch . 2011. “Effect Sizes in Experimental Pain Produced by Gender, Genetic Variants and Sensitization Procedures.” PLoS One 6: e17724. 21423693 10.1371/journal.pone.0017724 PMC3053372

[ejp70221-bib-0015] Firth, D. 1993. “Bias Reduction of Maximum Likelihood Estimates.” Biometrika 80: 27–38.

[ejp70221-bib-0016] Fonti, V. , and E. Belitser . 2017. “Feature Selection Using Lasso.” VU Amsterdam Research Paper in Business Analytics 30: 1–25.

[ejp70221-bib-0017] Fox, J. , and S. Weisberg . 2011. An R Companion to Applied Regression. 2nd ed. Sage.

[ejp70221-bib-0018] Friedman, J. , T. Hastie , and R. Tibshirani . 2010. “Regularization Paths for Generalized Linear Models via Coordinate Descent.” Journal of Statistical Software 33: 1–22. 20808728 PMC2929880

[ejp70221-bib-0019] Goreis, A. , S. Fanninger , A. Lozar , et al. 2025. “Water Temperature and Biological Sex Influence Cold Pressor Pain in Healthy Adults: A Randomized Within‐Subjects Trial.” Frontiers in Physiology 16: 1628111. 40740425 10.3389/fphys.2025.1628111 PMC12307380

[ejp70221-bib-0020] Gulati, M. , E. Dursun , K. Vincent , and F. E. Watt . 2023. “The Influence of Sex Hormones on Musculoskeletal Pain and Osteoarthritis.” Lancet Rheumatology 5: e225–e238. 38251525 10.1016/S2665-9913(23)00060-7

[ejp70221-bib-0021] Guyon, I. 2003. “An Introduction to Variable and Feature Selection.” Journal of Machine Learning Research 3: 1157–1182.

[ejp70221-bib-0022] Heston, T. F. , and J. M. King . 2017. “Predictive Power of Statistical Significance.” World Journal of Methodology 7: 112–116. 29354483 10.5662/wjm.v7.i4.112 PMC5746664

[ejp70221-bib-0023] Hoerl, A. E. , and R. W. Kennard . 1970. “Ridge Regression: Biased Estimation for Nonorthogonal Problems.” Technometrics 12: 55–67.

[ejp70221-bib-0024] Hohmann, E. , M. J. Wetzler , and R. B. D'Agostino . 2017. “Research Pearls: The Significance of Statistics and Perils of Pooling. Part 2: Predictive Modeling.” Arthroscopy 33: 1423–1432. 28457678 10.1016/j.arthro.2017.01.054 PMC7017839

[ejp70221-bib-0025] Hu, Y. H. , S. Palreddy , and W. J. Tompkins . 1997. “A Patient‐Adaptable ECG Beat Classifier Using a Mixture of Experts Approach.” IEEE Transactions on Biomedical Engineering 44: 891–900. 9282481 10.1109/10.623058

[ejp70221-bib-0026] Ihaka, R. , and R. Gentleman . 1996. “R: A Language for Data Analysis and Graphics.” Journal of Computational and Graphical Statistics 5: 299–314.

[ejp70221-bib-0027] Joharatnam, N. , D. F. McWilliams , D. Wilson , M. Wheeler , I. Pande , and D. A. Walsh . 2015. “A Cross‐Sectional Study of Pain Sensitivity, Disease‐Activity Assessment, Mental Health, and Fibromyalgia Status in Rheumatoid Arthritis.” Arthritis Research & Therapy 17: 11. 25600850 10.1186/s13075-015-0525-5 PMC4363056

[ejp70221-bib-0028] Karatzoglou, A. , A. Smola , K. Hornik , and A. Zeileis . 2004. “Kernlab—An S4 Package for Kernel Methods in R.” Journal of Statistical Software 11: 1–20.

[ejp70221-bib-0029] Keogh, E. 2022. “Sex and Gender Differences in Pain: Past, Present, and Future.” Pain 163: S108–S116. 36099334 10.1097/j.pain.0000000000002738

[ejp70221-bib-0030] Keogh, E. , and K. E. Boerner . 2024. “Challenges With Embedding an Integrated Sex and Gender Perspective Into Pain Research: Recommendations and Opportunities.” Brain, Behavior, and Immunity 117: 112–121. 38145854 10.1016/j.bbi.2023.12.027

[ejp70221-bib-0031] Khadirnaikar, S. , S. Shukla , and S. R. M. Prasanna . 2023. “Machine Learning Based Combination of Multi‐Omics Data for Subgroup Identification in Non‐Small Cell Lung Cancer.” Scientific Reports 13: 4636. 36944673 10.1038/s41598-023-31426-w PMC10030850

[ejp70221-bib-0032] Khalili, A. 2010. “New Estimation and Feature Selection Methods in Mixture‐Of‐Experts Models.” Canadian Journal of Statistics = La Revue Canadienne de Statistique 38: 519–539.

[ejp70221-bib-0033] Kilo, S. , M. Schmelz , M. Koltzenburg , and H. O. Handwerker . 1994. “Different Patterns of Hyperalgesia Induced by Experimental Inflammation in Human Skin.” Brain 117: 385–396. 8186964 10.1093/brain/117.2.385

[ejp70221-bib-0034] Kiso, T. , Y. Nagakura , T. Toya , et al. 2001. “Neurometer Measurement of Current Stimulus Threshold in Rats.” Journal of Pharmacology and Experimental Therapeutics 297: 352–356. 11259562

[ejp70221-bib-0035] Kohavi, R. 1995. “A Study of Cross‐Validation and Bootstrap for Accuracy Estimation and Model Selection.” In Proceedings of the 14th International Joint Conference on Artificial Intelligence, vol. 2, 1137–1143. Morgan Kaufmann Publishers Inc.

[ejp70221-bib-0036] Koltzenburg, M. , L. E. Lundberg , and H. E. Torebjork . 1992. “Dynamic and Static Components of Mechanical Hyperalgesia in Human Hairy Skin.” Pain 51: 207–219. 1484717 10.1016/0304-3959(92)90262-A

[ejp70221-bib-0037] Kondrats'kyĭ, A. P. , K. O. Kondrats'ka , R. Skryma , N. Prevars'ka , and I. M. Shuba . 2009. “Gender Differences in Cold Sensitivity: Role of Hormonal Regulation of TRPM8 Channel.” Fiziolohichnyĭ Zhurnal 55: 91–99. 19827635

[ejp70221-bib-0038] Kringel, D. , M. A. Kaunisto , E. Kalso , and J. Lötsch . 2019. “Machine‐Learned Analysis of Global and Glial/Opioid Intersection‐Related DNA Methylation in Patients With Persistent Pain After Breast Cancer Surgery.” Clinical Epigenetics 11: 167. 31775878 10.1186/s13148-019-0772-4 PMC6881976

[ejp70221-bib-0039] Kuhn, M. 2018. “caret: Classification and Regression Training.” https://github.com/topepo/caret/.

[ejp70221-bib-0040] Kuhn, M. , and R. Quinlan . 2018. “C50: C5.0 Decision Trees and Rule‐Based Models.” https://topepo.github.io/C5.0/.

[ejp70221-bib-0041] Kursa, M. B. , and W. R. Rudnicki . 2010. “Feature Selection With the Boruta Package.” Journal of Statistical Software 36: 13.

[ejp70221-bib-0042] Li, Y. , P. Wang , J. Xu , X. Shi , T. Yin , and F. Teng . 2024. “Noninvasive Radiomic Biomarkers for Predicting Pseudoprogression and Hyperprogression in Patients With Non‐Small Cell Lung Cancer Treated With Immune Checkpoint Inhibition.” Oncoimmunology 13: 2312628. 38343749 10.1080/2162402X.2024.2312628 PMC10857548

[ejp70221-bib-0043] Liaw, A. , and M. Wiener . 2002. “Classification and Regression by randomForest.” R News 2: 18–22.

[ejp70221-bib-0044] Liossi, C. , H. Laycock , K. Radhakrishnan , Z. Hussain , and D. E. Schoth . 2024. “A Systematic Review and Meta‐Analysis of Conditioned Pain Modulation in Children and Young People With Chronic Pain.” Children (Basel) 11: 11. 10.3390/children11111367 PMC11592744 39594942

[ejp70221-bib-0045] Lo, A. , H. Chernoff , T. Zheng , and S.‐H. Lo . 2015. “Why Significant Variables Aren't Automatically Good Predictors.” Proceedings of the National Academy of Sciences of the United States of America 112: 13892–13897. 26504198 10.1073/pnas.1518285112 PMC4653162

[ejp70221-bib-0046] Lötsch, J. , and M. S. Angst . 2003. “The Mu‐Opioid Agonist Remifentanil Attenuates Hyperalgesia Evoked by Blunt and Punctuated Stimuli With Different Potency: A Pharmacological Evaluation of the Freeze Lesion in Humans.” Pain 102: 151–161. 12620606 10.1016/s0304-3959(02)00349-4

[ejp70221-bib-0047] Lötsch, J. , K. Gasimli , S. Malkusch , et al. 2024. “Machine Learning and Biological Validation Identify Sphingolipids as Potential Mediators of Paclitaxel‐Induced Neuropathy in Cancer Patients.” eLife 13: 13. 10.7554/eLife.91941 PMC11444680 39347767

[ejp70221-bib-0048] Lötsch, J. , and S. Malkusch . 2021. “Interpretation of Cluster Structures in Pain‐Related Phenotype Data Using Explainable Artificial Intelligence (XAI).” European Journal of Pain 25: 442–465. 33064864 10.1002/ejp.1683

[ejp70221-bib-0049] Lötsch, J. , and B. Mayer . 2022. “A Biomedical Case Study Showing That Tuning Random Forests Can Fundamentally Change the Interpretation of Supervised Data Structure Exploration Aimed at Knowledge Discovery.” BioMedInformatics 2: 544–552.

[ejp70221-bib-0050] Lötsch, J. , B. Mayer , and D. Kringel . 2023. “Machine Learning Analysis Predicts a Person's Sex Based on Mechanical but Not Thermal Pain Thresholds.” Scientific Reports 13: 7332. 37147321 10.1038/s41598-023-33337-2 PMC10163041

[ejp70221-bib-0051] Lötsch, J. , M. Rohrbacher , H. Schmidt , A. Doehring , J. Brockmöller , and G. Geisslinger . 2009. “Can Extremely Low or High Morphine Formation From Codeine Be Predicted Prior to Therapy Initiation?” Pain 144: 119–124. 19395173 10.1016/j.pain.2009.03.023

[ejp70221-bib-0052] Lotsch, J. , and A. Ultsch . 2017. “Machine Learning in Pain Research.” Pain 159: 623–630. 10.1097/j.pain.0000000000001118 PMC5895117 29194126

[ejp70221-bib-0053] Lötsch, J. , and A. Ultsch . 2022. “Enhancing Explainable Machine Learning by Reconsidering Initially Unselected Items in Feature Selection for Classification.” BioMedInformatics 2: 701–714.

[ejp70221-bib-0054] Lötsch, J. , and A. Ultsch . 2023a. “Pitfalls of Using Multinomial Regression Analysis to Identify Class‐Structure‐Relevant Variables in Biomedical Data Sets: Why a Mixture of Experts (MOE) Approach Is Better.” BioMedInformatics 3: 869–884.

[ejp70221-bib-0055] Lötsch, J. , and A. Ultsch . 2023b. “Recursive Computed ABC (cABC) Analysis as a Precise Method for Reducing Machine Learning Based Feature Sets to Their Minimum Informative Size.” Scientific Reports 13: 5470. 37016033 10.1038/s41598-023-32396-9 PMC10073099

[ejp70221-bib-0056] Lötsch, J. , A. Ultsch , and E. Kalso . 2017. “Prediction of Persistent Post‐Surgery Pain by Preoperative Cold Pain Sensitivity: Biomarker Development With Machine‐Learning‐Derived Analysis.” British Journal of Anaesthesia 119: 821–829. 29121286 10.1093/bja/aex236

[ejp70221-bib-0057] Lötsch, J. , A. Ultsch , B. Mayer , and D. Kringel . 2022. “Artificial Intelligence and Machine Learning in Pain Research: A Data Scientometric Analysis.” PAIN Reports 7: e1044. 36348668 10.1097/PR9.0000000000001044 PMC9635040

[ejp70221-bib-0058] Marquardt, D. W. 1970. “Generalized Inverses, Ridge Regression, Biased Linear Estimation, and Nonlinear Estimation.” Technometrics 12: 591–612.

[ejp70221-bib-0059] Martina Udovicic, K. B. L. B.‐Z. M. P. 2007. “What We Need to Know When Calculating the Coefficient of Correlation?” Biochemia Medica 17: 10–15.

[ejp70221-bib-0060] Masson, E. A. , A. Veves , D. Fernando , and A. J. Boulton . 1989. “Current Perception Thresholds: A New, Quick, and Reproducible Method for the Assessment of Peripheral Neuropathy in Diabetes Mellitus.” Diabetologia 32: 724–728. 2591640 10.1007/BF00274531

[ejp70221-bib-0061] Mease, P. J. 2011. “Measures of Psoriatic Arthritis: Tender and Swollen Joint Assessment, Psoriasis Area and Severity Index (PASI), Nail Psoriasis Severity Index (NAPSI), Modified Nail Psoriasis Severity Index (mNAPSI), Mander/Newcastle Enthesitis Index (MEI), Leeds Enthesitis Index (LEI), Spondyloarthritis Research Consortium of Canada (SPARCC), Maastricht Ankylosing Spondylitis Enthesis Score (MASES), Leeds Dactylitis Index (LDI), Patient Global for Psoriatic Arthritis, Dermatology Life Quality Index (DLQI), Psoriatic Arthritis Quality of Life (PsAQOL), Functional Assessment of Chronic Illness Therapy‐Fatigue (FACIT‐F), Psoriatic Arthritis Response Criteria (PsARC), Psoriatic Arthritis Joint Activity Index (PsAJAI), Disease Activity in Psoriatic Arthritis (DAPSA), and Composite Psoriatic Disease Activity Index (CPDAI).” Arthritis Care & Research (Hoboken) 63, no. Suppl 11: S64–S85. 10.1002/acr.20577 22588772

[ejp70221-bib-0062] Metropolis, N. , and S. Ulam . 1949. “The Monte Carlo Method.” Journal of the American Statistical Association 44: 335–341. 18139350 10.1080/01621459.1949.10483310

[ejp70221-bib-0063] Minsky, M. , and S. Papert . 1969. Perceptrons; an Introduction to Computational Geometry. MIT Press.

[ejp70221-bib-0064] Mogil, J. S. 2012. “Sex Differences in Pain and Pain Inhibition: Multiple Explanations of a Controversial Phenomenon.” Nature Reviews. Neuroscience 13: 859–866. 23165262 10.1038/nrn3360

[ejp70221-bib-0065] Mogil, J. S. 2020. “Qualitative Sex Differences in Pain Processing: Emerging Evidence of a Biased Literature.” Nature Reviews. Neuroscience 21: 353–365. 32440016 10.1038/s41583-020-0310-6

[ejp70221-bib-0066] Murphy, K. P. 2012. Machine Learning: A Probabilistic Perspective. MIT Press.

[ejp70221-bib-0067] Osborne, N. R. , and K. D. Davis . 2022. “Sex and Gender Differences in Pain.” International Review of Neurobiology 164: 277–307. 36038207 10.1016/bs.irn.2022.06.013

[ejp70221-bib-0068] Pitei, D. L. , P. J. Watkins , M. J. Stevens , and M. E. Edmonds . 1994. “The Value of the Neurometer in Assessing Diabetic Neuropathy by Measurement of the Current Perception Threshold.” Diabetic Medicine 11: 872–876. 7705025 10.1111/j.1464-5491.1994.tb00371.x

[ejp70221-bib-0069] Pradier, M. F. , J. Zazo , S. Parbhoo , R. H. Perlis , M. Zazzi , and F. Doshi‐Velez . 2021. “Preferential Mixture‐Of‐Experts: Interpretable Models That Rely on Human Expertise as Much as Possible.” AMIA Summits on Translational Science Proceedings 2021: 525–534. PMC8378634 34457168

[ejp70221-bib-0070] Quinlan, J. R. 1986. “Induction of Decision Trees.” Machine Learning 1: 81–106.

[ejp70221-bib-0071] Rischke, S. , S. M. Poor , R. Gurke , et al. 2023. “Machine Learning Identifies Right Index Finger Tenderness as Key Signal of DAS28‐CRP Based Psoriatic Arthritis Activity.” Scientific Reports 13: 22710. 38123604 10.1038/s41598-023-49574-4 PMC10733369

[ejp70221-bib-0072] Robin, X. , N. Turck , A. Hainard , et al. 2011. “pROC: An Open‐Source Package for R and S+ to Analyze and Compare ROC Curves.” BMC Bioinformatics 12: 77. 21414208 10.1186/1471-2105-12-77 PMC3068975

[ejp70221-bib-0073] Robinson, M. E. , E. A. Wise , I. J. L. Riley , and J. W. Atchison . 1998. “Sex Differences in Clinical Pain: A Multisample Study.” Journal of Clinical Psychology in Medical Settings 5: 413–424.

[ejp70221-bib-0074] Rosenblatt, F. 1958. “The Perceptron: A Probabilistic Model for Information Storage and Organization in the Brain.” Psychological Review 65: 386–408. 13602029 10.1037/h0042519

[ejp70221-bib-0075] Rügamer, D. , F. Pfisterer , B. Bischl , and B. Grün . 2024. “Mixture of Experts Distributional Regression: Implementation Using Robust Estimation With Adaptive First‐Order Methods.” AStA Advances in Statistical Analysis 108: 351–373.

[ejp70221-bib-0076] Shalev‐Shwartz, S. , and S. Ben‐David . 2014. Understanding Machine Learning: From Theory to Algorithms. Cambridge University Press.

[ejp70221-bib-0077] Shin, S. M. , F. Moehring , B. Itson‐Zoske , et al. 2021. “Piezo2 Mechanosensitive Ion Channel Is Located to Sensory Neurons and Nonneuronal Cells in Rat Peripheral Sensory Pathway: Implications in Pain.” Pain 162: 2750–2768. 34285153 10.1097/j.pain.0000000000002356 PMC8526381

[ejp70221-bib-0078] Sievert, C. 2020. Interactive Web‐Based Data Visualization With R, Plotly, and Shiny. Chapman and Hall/CRC.

[ejp70221-bib-0079] Singh, H. , S. Arora , V. Tanwar , A. Kalra , G. Sukhija , and N. Govil . 2020. “The Validity and Sensitivity of Rheumatoid Arthritis Pain Scale on a Different Ethnic Group From Indian Rheumatoid Arthritis Patients.” Archives of Rheumatology 35: 90–96. 32637924 10.5606/ArchRheumatol.2020.7348 PMC7322297

[ejp70221-bib-0080] Singh, J. A. , K. G. Saag , S. L. Bridges Jr. , et al. 2016. “2015 American College of Rheumatology Guideline for the Treatment of Rheumatoid Arthritis.” Arthritis Care & Research (Hoboken) 68: 1–25. 10.1002/acr.22783 26545825

[ejp70221-bib-0081] Sisignano, M. , J. Lotsch , M. J. Parnham , and G. Geisslinger . 2019. “Potential Biomarkers for Persistent and Neuropathic Pain Therapy.” Pharmacology & Therapeutics 199: 16–29. 30759376 10.1016/j.pharmthera.2019.02.004

[ejp70221-bib-0082] Sorge, R. E. , and S. K. Totsch . 2017. “Sex Differences in Pain.” Journal of Neuroscience Research 95: 1271–1281. 27452349 10.1002/jnr.23841

[ejp70221-bib-0083] Student . 1908. “The Probable Error of a Mean.” Biometrika 6: 1–25.

[ejp70221-bib-0084] Takefuji, Y. 2025. “Limitations of Logistic Regression in Analyzing Complex Ambulatory Blood Pressure Data: A Call for Non‐Parametric Approaches.” European Heart Journal 46: 3790–3791. 40717668 10.1093/eurheartj/ehaf541

[ejp70221-bib-0085] Tay, J. K. , B. Narasimhan , and T. Hastie . 2023. “Elastic Net Regularization Paths for All Generalized Linear Models.” Journal of Statistical Software 106: 1–31. 37138589 10.18637/jss.v106.i01 PMC10153598

[ejp70221-bib-0086] Tibshirani, R. 1996. “Regression Shrinkage and Selection via the Lasso.” Journal of the Royal Statistical Society. Series B, Statistical Methodology 58: 267–288.

[ejp70221-bib-0087] Ultsch, A. , and J. Lötsch . 2020. “The Fundamental Clustering and Projection Suite (FCPS): A Dataset Collection to Test the Performance of Clustering and Data Projection Algorithms.” Data 5: 13.

[ejp70221-bib-0088] Varga, T. V. , K. Niss , A. C. Estampador , C. B. Collin , and P. L. Moseley . 2020. “Association Is Not Prediction: A Landscape of Confused Reporting in Diabetes—A Systematic Review.” Diabetes Research and Clinical Practice 170: 108497. 33068662 10.1016/j.diabres.2020.108497

[ejp70221-bib-0089] Vasconcelos, D. P. , C. Jabangwe , M. Lamghari , and C. J. Alves . 2022. “The Neuroimmune Interplay in Joint Pain: The Role of Macrophages.” Frontiers in Immunology 13: 812962. 35355986 10.3389/fimmu.2022.812962 PMC8959978

[ejp70221-bib-0090] Venables, W. N. , and B. D. Ripley . 2002. Modern Applied Statistics With S. Springer.

[ejp70221-bib-0091] Veves, A. , M. J. Young , C. Manes , and A. J. Boulton . 1994. “Differences in Peripheral and Autonomic Nerve Function Measurements in Painful and Painless Neuropathy. A Clinical Study.” Diabetes Care 17: 1200–1202. 7821144 10.2337/diacare.17.10.1200

[ejp70221-bib-0092] Wallace, M. S. , J. B. Dyck , S. S. Rossi , and T. L. Yaksh . 1996. “Computer‐Controlled Lidocaine Infusion for the Evaluation of Neuropathic Pain After Peripheral Nerve Injury.” Pain 66: 69–77. 8857633 10.1016/0304-3959(96)02980-6

[ejp70221-bib-0093] Ward, J. J. H. 1963. “Hierarchical Grouping to Optimize an Objective Function.” Journal of the American Statistical Association 58: 236–244.

[ejp70221-bib-0094] Wickham, H. 2009. ggplot2: Elegant Graphics for Data Analysis. Springer‐Verlag.

[ejp70221-bib-0095] Zou, H. , and T. Hastie . 2005. “Regularization and Variable Selection via the Elastic Net.” Journal of the Royal Statistical Society. Series B, Statistical Methodology 67: 301–320.

