# Supplementary material for: Resolving Interpretation Challenges in Machine Learning Feature Selection With an Iterative Approach in Biomedical Pain Data
Source: Eur J Pain. 2026 Jan 26;30(2):e70221. doi: 10.1002/ejp.70221 (PMC12834868; doi:10.1002/ejp.70221)
Supplement: Supplementary file 1 — Data S1: ejp70221‐sup‐0001‐Supinfo.pdf. [file EJP-30-0-s001.pdf]

Supplementary Tables and Figures

Supplementary Tables

Supplementary Table 1: Results of the analyses applying the current framework. The final selected features represent the minimal sufficient set identified through a four-phase iterative process combining backward elimination and individual rescue testing. The final rejected features are those that neither contributed to robust classification nor demonstrated independent predictive ability.

**Data set**	**Features**	
**“pain_thresholds_sex”**	FINAL SELECTED FEATURES: 3	Pressure2, Pressure, Current
	FINAL REJECTED FEATURES: 9	Heat, Heat_Capsaicin, Capsaicin_Effect_Heat, Cold, Cold_Menthol, Menthol_Effect_Cold, vonFrey, vonFrey_Capsaicin, Capsaicin_Effect_vonFrey
**“PsA DAS28-CRP"**	FINAL SELECTED FEATURES: 4	right_index_finger_pip_tender, visit_da_sga, right_ring_finger_pip_tender, right_middle_finger_pip_tender
	FINAL REJECTED FEATURES: 54	visit_da_crp, right_shoulder_swollen, left_shoulder_swollen, right_elbow_swollen, left_elbow_swollen, right_wrist_swollen, left_wrist_swollen, right_thumb_mcp_swollen, left_thumb_mcp_swollen, right_thumb_pip_swollen, left_thumb_pip_swollen, right_pinky_little_finger_mcp_swollen, right_ring_finger_mcp_swollen, right_middle_finger_mcp_swollen, right_index_finger_mcp_swollen, left_index_finger_mcp_swollen, left_middle_finger_mcp_swollen, left_ring_finger_mcp_swollen, left_pinky_little_finger_mcp_swollen, right_pinky_little_finger_pip_swollen, right_ring_finger_pip_swollen,

		right_middle_finger_pip_swollen, right_index_finger_pip_swollen, left_index_finger_pip_swollen, left_middle_finger_pip_swollen, left_ring_finger_pip_swollen, left_pinky_little_finger_pip_swollen, right_knee_swollen, left_knee_swollen, right_shoulder_tender, left_shoulder_tender, right_elbow_tender, left_elbow_tender, right_wrist_tender, left_wrist_tender, right_thumb_mcp_tender, left_thumb_mcp_tender, right_thumb_pip_tender, left_thumb_pip_tender, right_pinky_little_finger_mcp_tender, right_ring_finger_mcp_tender, right_middle_finger_mcp_tender, right_index_finger_mcp_tender, left_index_finger_mcp_tender, left_middle_finger_mcp_tender, left_ring_finger_mcp_tender, left_pinky_little_finger_mcp_tender, right_pinky_little_finger_pip_tender, left_index_finger_pip_tender, left_middle_finger_pip_tender, left_ring_finger_pip_tender, left_pinky_little_finger_pip_tender, right_knee_tender, left_knee_tender

Supplementary Table 2: Results of standard (for comparison) and penalized logistic regression models predicting group membership. For each predictor, the table shows the coefficient from the unpenalized logistic regression (glm_estimate) and its Wald test p value (glm_p), together with coefficients from ridge (ridge_coef), lasso (lasso_coef), and elastic net (elastic_coef, α=0.5) models, all on the log-odds scale. Ridge selection (ridge_selected) is defined as an absolute ridge coefficient greater than 0.05, whereas lasso_selected and elastic_selected indicate non-zero coefficients in the corresponding penalized models. Rows with missing glm_estimate and glm_p correspond to predictors omitted from the standard model (e.g. due to singularities) but retained in the penalized models.

Variable	glm_p	ridge_coef	ridge_selected	lasso_coef	lasso_selected	elastic_coef	elastic_selected
**"pain_thresholds_sex" dataset**							
Capsaicin_Effect_Heat	NA	-0.032	FALSE	0.000	FALSE	0.000	FALSE
Capsaicin_Effect_vonFrey	NA	0.004	FALSE	0.000	FALSE	0.000	FALSE
Cold	0.472	-0.002	FALSE	0.000	FALSE	0.000	FALSE
Cold_Menthol	0.203	-0.059	TRUE	0.000	FALSE	0.000	FALSE
**Current**	0.031	-0.214	TRUE	-0.186	TRUE	-0.199	TRUE
Heat	0.362	0.036	FALSE	0.000	FALSE	0.000	FALSE
Heat_Capsaicin	0.296	0.047	FALSE	0.000	FALSE	0.000	FALSE
Menthol_Effect_Cold	NA	0.095	TRUE	0.000	FALSE	0.003	TRUE
**Pressure**	0.724	-0.230	TRUE	-0.538	TRUE	-0.291	TRUE
**Pressure2**	0.769	-0.226	TRUE	0.000	FALSE	-0.231	TRUE
vonFrey	0.739	-0.068	TRUE	0.000	FALSE	0.000	FALSE
vonFrey_Capsaicin	0.615	-0.076	TRUE	0.000	FALSE	0.000	FALSE
**"PsA DAS28-CRP" dataset**							
left_elbow_swollen	NA	0.000	FALSE	0.000	FALSE	0.000	FALSE
left_elbow_tender	NA	-0.158	TRUE	0.000	FALSE	0.000	FALSE
left_index_finger_mcp_swollen	NA	0.000	FALSE	0.000	FALSE	0.000	FALSE
left_index_finger_mcp_tender	NA	1.714	TRUE	2.043	TRUE	2.850	TRUE
left_index_finger_pip_swollen	NA	0.000	FALSE	0.000	FALSE	0.000	FALSE
left_index_finger_pip_tender	1.000	0.460	TRUE	0.000	FALSE	0.000	FALSE
left_knee_swollen	NA	0.000	FALSE	0.000	FALSE	0.000	FALSE
left_knee_tender	NA	0.000	FALSE	0.000	FALSE	0.000	FALSE
left_middle_finger_mcp_swollen	NA	0.000	FALSE	0.000	FALSE	0.000	FALSE
left_middle_finger_mcp_tender	NA	0.689	TRUE	0.000	FALSE	0.000	FALSE
left_middle_finger_pip_swollen	NA	0.000	FALSE	0.000	FALSE	0.000	FALSE
left_middle_finger_pip_tender	NA	0.002	FALSE	0.000	FALSE	0.000	FALSE
left_pinky_little_finger_mcp_swollen	NA	0.000	FALSE	0.000	FALSE	0.000	FALSE
left_pinky_little_finger_mcp_tender	NA	0.000	FALSE	0.000	FALSE	0.000	FALSE
left_pinky_little_finger_pip_swollen	NA	0.000	FALSE	0.000	FALSE	0.000	FALSE
left_pinky_little_finger_pip_tender	NA	0.002	FALSE	0.000	FALSE	0.000	FALSE
left_ring_finger_mcp_swollen	NA	0.000	FALSE	0.000	FALSE	0.000	FALSE
left_ring_finger_mcp_tender	NA	1.953	TRUE	0.051	TRUE	2.065	TRUE
left_ring_finger_pip_swollen	NA	0.000	FALSE	0.000	FALSE	0.000	FALSE
left_ring_finger_pip_tender	1.000	0.262	TRUE	0.000	FALSE	0.000	FALSE
left_shoulder_swollen	NA	0.000	FALSE	0.000	FALSE	0.000	FALSE
left_shoulder_tender	NA	0.000	FALSE	0.000	FALSE	0.000	FALSE
left_thumb_mcp_swollen	NA	0.000	FALSE	0.000	FALSE	0.000	FALSE
left_thumb_mcp_tender	1.000	0.182	TRUE	0.000	FALSE	0.000	FALSE
left_thumb_pip_swollen	NA	0.000	FALSE	0.000	FALSE	0.000	FALSE
left_thumb_pip_tender	NA	0.002	FALSE	0.000	FALSE	0.000	FALSE
left_wrist_swollen	NA	0.000	FALSE	0.000	FALSE	0.000	FALSE

left_wrist_tender	1.000	2.660	TRUE	1.730	TRUE	3.331	TRUE
right_elbow_swollen	NA	0.000	FALSE	0.000	FALSE	0.000	FALSE
right_elbow_tender	1.000	-0.158	TRUE	0.000	FALSE	0.000	FALSE
right_index_finger_mcp_swollen	1.000	2.696	TRUE	0.827	TRUE	2.415	TRUE
right_index_finger_mcp_tender	NA	0.689	TRUE	0.000	FALSE	0.000	FALSE
right_index_finger_pip_swollen	1.000	1.386	TRUE	0.000	FALSE	0.851	TRUE
**right_index_finger_pip_tender**	0.999	3.169	TRUE	4.208	TRUE	5.383	TRUE
right_knee_swollen	NA	0.000	FALSE	0.000	FALSE	0.000	FALSE
right_knee_tender	1.000	0.657	TRUE	0.000	FALSE	0.000	FALSE
right_middle_finger_mcp_swollen	1.000	-0.386	TRUE	0.000	FALSE	0.000	FALSE
right_middle_finger_mcp_tender	1.000	0.688	TRUE	0.000	FALSE	0.000	FALSE
right_middle_finger_pip_swollen	NA	-0.385	TRUE	0.000	FALSE	0.000	FALSE
**right_middle_finger_pip_tender**	0.999	3.254	TRUE	3.101	TRUE	5.034	TRUE
right_pinky_little_finger_mcp_swollen	NA	0.000	FALSE	0.000	FALSE	0.000	FALSE
right_pinky_little_finger_mcp_tender	1.000	1.953	TRUE	2.045	TRUE	2.063	TRUE
right_pinky_little_finger_pip_swollen	NA	0.000	FALSE	0.000	FALSE	0.000	FALSE
right_pinky_little_finger_pip_tender	1.000	1.109	TRUE	0.000	FALSE	0.000	FALSE
right_ring_finger_mcp_swollen	NA	0.000	FALSE	0.000	FALSE	0.000	FALSE
right_ring_finger_mcp_tender	NA	0.000	FALSE	0.000	FALSE	0.000	FALSE
right_ring_finger_pip_swollen	NA	0.000	FALSE	0.000	FALSE	0.000	FALSE
**right_ring_finger_pip_tender**	1.000	2.953	TRUE	2.955	TRUE	4.563	TRUE
right_shoulder_swollen	NA	0.000	FALSE	0.000	FALSE	0.000	FALSE
right_shoulder_tender	1.000	4.604	TRUE	4.061	TRUE	6.523	TRUE
right_thumb_mcp_swollen	1.000	-1.087	TRUE	0.000	FALSE	0.000	FALSE
right_thumb_mcp_tender	1.000	0.867	TRUE	0.722	TRUE	1.398	TRUE
right_thumb_pip_swollen	NA	0.000	FALSE	0.000	FALSE	0.000	FALSE
right_thumb_pip_tender	1.000	0.002	FALSE	0.000	FALSE	0.000	FALSE
right_wrist_swollen	NA	0.000	FALSE	0.000	FALSE	0.000	FALSE
right_wrist_tender	1.000	1.299	TRUE	1.201	TRUE	1.840	TRUE
visit_da_crp	1.000	0.096	TRUE	0.062	TRUE	0.133	TRUE
**visit_da_sga**	0.999	0.057	TRUE	0.071	TRUE	0.104	TRUE

Supplementary Table 3: PsA DAS28-CRP dataset: Logistic regression results for the full set of variables and after removal of variables with high variance inflation factors (VIF). For each predictor, regression coefficients (Estimate), standard errors, test statistics (z value), and corresponding p-values (Pr(>|z|)) are presented, with conventional significance markers (p < 0.05; *p < 0.01; **p < 0.001; - not significant). Predictors associated with structural collinearity yielded missing values (NA).

**Variables**	**PsA dataset; all variables**					**PsA dataset; VIF removed**				
	Estimate	Std. Error	z value	Pr(>|z|)	Significance	Estimate	Std. Error	z value	Pr(>|z|)	Significance
**(Intercept)**	-2.07E+02	1.34E+05	-0.002	0.999	-	-2.07E+02	1.34E+05	-0.002	0.999	
**visit_da_crp**	8.74E-01	2.48E+04	0	1	-	8.74E-01	2.48E+04	0	1	
**visit_da_sga**	2.81E+00	1.63E+03	0.002	0.999	-	2.81E+00	1.63E+03	0.002	0.999	
**right_shoulder_swollen**	NA	NA	NA	NA	-					
**left_shoulder_swollen**	NA	NA	NA	NA	-					
**right_elbow_swollen**	NA	NA	NA	NA	-					
**left_elbow_swollen**	NA	NA	NA	NA	-					
**right_wrist_swollen**	NA	NA	NA	NA	-					
**left_wrist_swollen**	NA	NA	NA	NA	-					
**right_thumb_mcp_swollen**	6.80E+01	4.58E+05	0	1	-	6.80E+01	4.58E+05	0	1	
**left_thumb_mcp_swollen**	NA	NA	NA	NA	-					
**right_thumb_pip_swollen**	NA	NA	NA	NA	-					
**left_thumb_pip_swollen**	NA	NA	NA	NA	-					
**right_pinky_little_finger_mcp_swollen**	NA	NA	NA	NA	-					
**right_ring_finger_mcp_swollen**	NA	NA	NA	NA	-					
**right_middle_finger_mcp_swollen**	6.45E+01	5.33E+05	0	1	-	6.45E+01	5.33E+05	0	1	
**right_index_finger_mcp_swollen**	6.35E+01	3.61E+05	0	1	-	6.35E+01	3.61E+05	0	1	
**left_index_finger_mcp_swollen**	NA	NA	NA	NA	-					
**left_middle_finger_mcp_swollen**	NA	NA	NA	NA	-					
**left_ring_finger_mcp_swollen**	NA	NA	NA	NA	-					
**left_pinky_little_finger_mcp_swollen**	NA	NA	NA	NA	-					
**right_pinky_little_finger_pip_swollen**	NA	NA	NA	NA	-					
**right_ring_finger_pip_swollen**	NA	NA	NA	NA	-					
**right_middle_finger_pip_swollen**	NA	NA	NA	NA	-					
**right_index_finger_pip_swollen**	1.34E+01	2.75E+05	0	1	-	1.34E+01	2.75E+05	0	1	
**left_index_finger_pip_swollen**	NA	NA	NA	NA	-					
**left_middle_finger_pip_swollen**	NA	NA	NA	NA	-					

**left_ring_finger_pip_swollen**	NA	NA	NA	NA	-					
**left_pinky_little_finger_pip_swollen**	NA	NA	NA	NA	-					
**right_knee_swollen**	NA	NA	NA	NA	-					
**left_knee_swollen**	NA	NA	NA	NA	-					
**right_shoulder_tender**	1.16E+02	3.62E+05	0	1	-	1.16E+02	3.62E+05	0	1	
**left_shoulder_tender**	NA	NA	NA	NA	-					
**right_elbow_tender**	1.78E+02	3.75E+05	0	1	-	1.78E+02	3.75E+05	0	1	
**left_elbow_tender**	NA	NA	NA	NA	-					
**right_wrist_tender**	7.67E+01	8.30E+05	0	1	-	7.67E+01	8.30E+05	0	1	
**left_wrist_tender**	3.46E+01	5.37E+05	0	1	-	3.46E+01	5.37E+05	0	1	
**right_thumb_mcp_tender**	8.95E+01	8.06E+05	0	1	-	8.95E+01	8.06E+05	0	1	
**left_thumb_mcp_tender**	6.04E+01	4.75E+07	0	1	-	6.04E+01	4.75E+07	0	1	
**right_thumb_pip_tender**	9.33E+00	4.75E+07	0	1	-	9.33E+00	4.75E+07	0	1	
**left_thumb_pip_tender**	NA	NA	NA	NA	-					
**right_pinky_little_finger_mcp_tender**	-2.59E+01	4.75E+07	0	1	-	-2.59E+01	4.75E+07	0	1	
**right_ring_finger_mcp_tender**	NA	NA	NA	NA	-					
**right_middle_finger_mcp_tender**	4.38E+01	5.06E+05	0	1	-	4.38E+01	5.06E+05	0	1	
**right_index_finger_mcp_tender**	NA	NA	NA	NA	-					
**left_index_finger_mcp_tender**	NA	NA	NA	NA	-					
**left_middle_finger_mcp_tender**	NA	NA	NA	NA	-					
**left_ring_finger_mcp_tender**	NA	NA	NA	NA	-					
**left_pinky_little_finger_mcp_tender**	NA	NA	NA	NA	-					
**right_pinky_little_finger_pip_tender**	-7.42E+01	9.53E+05	0	1	-	-7.42E+01	9.53E+05	0	1	
**right_ring_finger_pip_tender**	6.22E+01	3.57E+05	0	1	-	6.22E+01	3.57E+05	0	1	
**right_middle_finger_pip_tender**	8.74E+01	1.38E+05	0.001	0.999	-	8.74E+01	1.38E+05	0.001	0.999	
**right_index_finger_pip_tender**	1.02E+02	1.60E+05	0.001	0.999	-	1.02E+02	1.60E+05	0.001	0.999	
**left_index_finger_pip_tender**	3.53E+01	4.75E+07	0	1	-	3.53E+01	4.75E+07	0	1	
**left_middle_finger_pip_tender**	NA	NA	NA	NA	-					
**left_ring_finger_pip_tender**	-1.86E+02	1.00E+06	0	1	-	-1.86E+02	1.00E+06	0	1	
**left_pinky_little_finger_pip_tender**	NA	NA	NA	NA	-					
**right_knee_tender**	-4.47E+01	6.69E+05	0	1	-	-4.47E+01	6.69E+05	0	1	
**left_knee_tender**	NA	NA	NA	NA	-					

Supplementary Figures

Supplementary Figure 1: Artificial “FCPS::Atom” dataset: Three-dimensional scatter plot of the Fundamental Clustering and Projection Suite (FCPS) dataset “Atom” (Ultsch and Lötsch 2020) visualizing the distribution of observations across its three numeric features. Points are colored by class membership, facilitating visual assessment of class separation in the feature space.

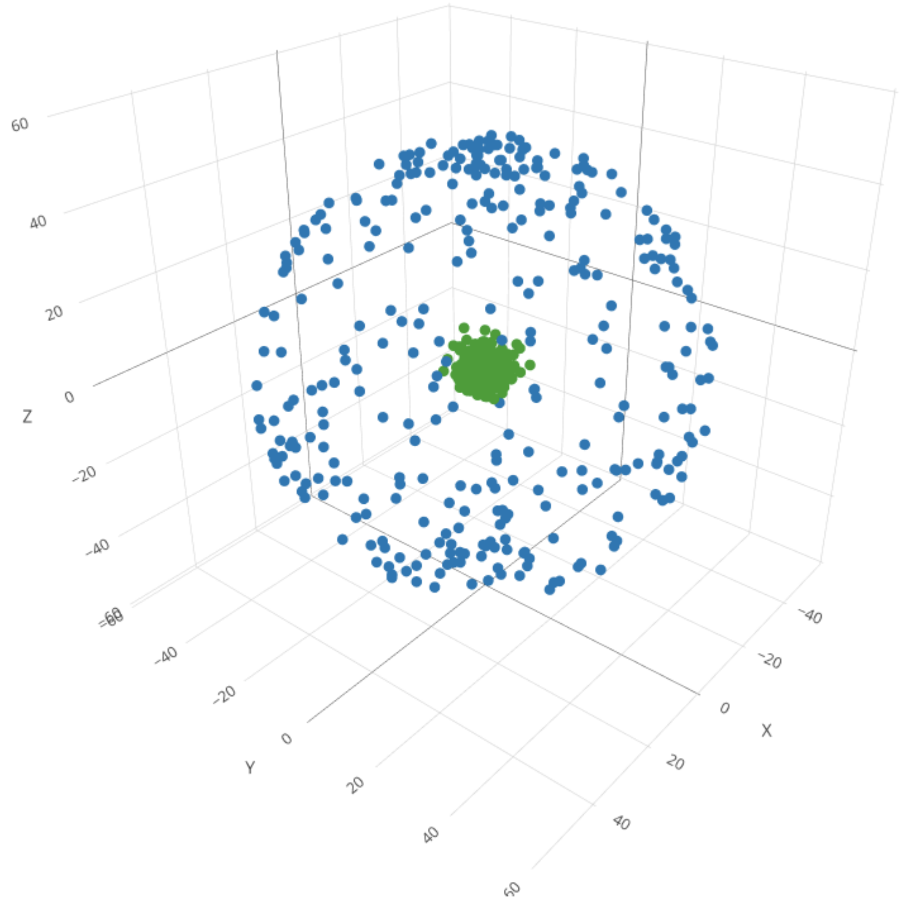

References

Ultsch A and Lötsch J. The Fundamental Clustering and Projection Suite (FCPS): A Dataset Collection to Test the Performance of Clustering and Data Projection Algorithms. Data 2020;5: 13.
